# Supramolecular polymers form tactoids through liquid–liquid phase separation

**DOI:** 10.1038/s41586-024-07034-7

**Published:** 2024-02-28

**Authors:** Hailin Fu, Jingyi Huang, Joost J. B. van der Tol, Lu Su, Yuyang Wang, Swayandipta Dey, Peter Zijlstra, George Fytas, Ghislaine Vantomme, Patricia Y. W. Dankers, E. W. Meijer

**Affiliations:** 1 Institute for Complex Molecular Systems, Eindhoven University of Technology, Eindhoven, The Netherlands; 2 Department of Chemistry and Chemical Engineering and Laboratory of Macromolecular and Organic Chemistry, Eindhoven University of Technology, Eindhoven, The Netherlands; 3 Department of Biomedical Engineering and Laboratory of Chemical Biology, Eindhoven University of Technology, Eindhoven, The Netherlands; 4 Leiden Academic Centre for Drug Research, Leiden University, Leiden, The Netherlands; 5 Department of Applied Physics and Science Education, Eindhoven University of Technology, Eindhoven, The Netherlands; 6 Eindhoven Hendrik Casimir Institute, Eindhoven University of Technology, Eindhoven, The Netherlands; 7 Max Planck Institute for Polymer Research, Mainz, Germany; 8 Institute of Electronic Structure and Laser, FO.R.T.H, Heraklion, Greece; 9 School of Chemistry and RNA Institute, University of New South Wales, Sydney, New South Wales Australia

**Keywords:** Supramolecular polymers, Self-assembly

## Abstract

Liquid–liquid phase separation (LLPS) of biopolymers has recently been shown to play a central role in the formation of membraneless organelles with a multitude of biological functions^1–3^. The interplay between LLPS and macromolecular condensation is part of continuing studies^4,5^. Synthetic supramolecular polymers are the non-covalent equivalent of macromolecules but they are not reported to undergo LLPS yet. Here we show that continuously growing fibrils, obtained from supramolecular polymerizations of synthetic components, are responsible for phase separation into highly anisotropic aqueous liquid droplets (tactoids) by means of an entropy-driven pathway. The crowding environment, regulated by dextran concentration, affects not only the kinetics of supramolecular polymerizations but also the properties of LLPS, including phase-separation kinetics, morphology, internal order, fluidity and mechanical properties of the final tactoids. In addition, substrate–liquid and liquid–liquid interfaces proved capable of accelerating LLPS of supramolecular polymers, allowing the generation of a myriad of three-dimensional-ordered structures, including highly ordered arrays of micrometre-long tactoids at surfaces. The generality and many possibilities of supramolecular polymerizations to control emerging morphologies are demonstrated with several supramolecular polymers, opening up a new field of matter ranging from highly structured aqueous solutions by means of stabilized LLPS to nanoscopic soft matter.

## Abstract (web summary)

Spontaneous liquid–liquid phase-separation behaviour of high-aspect-ratio fibrils, obtained from supramolecular polymerizations of synthetic components, forms tactoids by means of an entropy-driven pathway.

## Main

Supramolecular polymers are known to form homogeneous solutions and gels under dilute and concentrated conditions, respectively, or precipitate if they are not sufficiently soluble in the solvent used^6,7^. By contrast, macromolecules can also undergo liquid–liquid phase separation (LLPS) and form inhomogeneous solutions with spherical droplets^8–11^. Condensations of biomacromolecules can lead to LLPS; however, the formation of droplets can also cause further condensations or aggregations of biomacromolecules^1,4^. Although there are reports of LLPS of small organic molecules preceding the formation of supramolecular polymers or crystals, synthetic supramolecular polymers undergoing LLPS has not been reported yet^12,13^. This is even more surprising given the similarities of supramolecular polymers to biological fibrils and their enormous potential for biomedical applications, either as solid materials or as drug-assembled nanostructures and biofunctional hydrogels^14–18^. Biofilaments such as actin, amyloid fibrils, microtubules, collagen and nanocellulose can form structured liquids or liquid crystals in vitro^19–25^. According to Onsager’s theory, phase separation and spontaneous ordering of rod-like colloids are largely driven by maximization of the translational entropy at high concentrations^26^. This theory is beautifully adopted in the liquid crystal field to explain the internal ordering of liquid crystalline phases^27^. Macromolecular crowders have been reported to be promotional in the phase separation of colloids and proteins and in the formation of coacervates and biomolecular condensates through volume exclusion effect^28–32^. Recently it has also been shown that macromolecular crowders could influence the supramolecular polymerization process^33–35^.

**Fig. 1:**
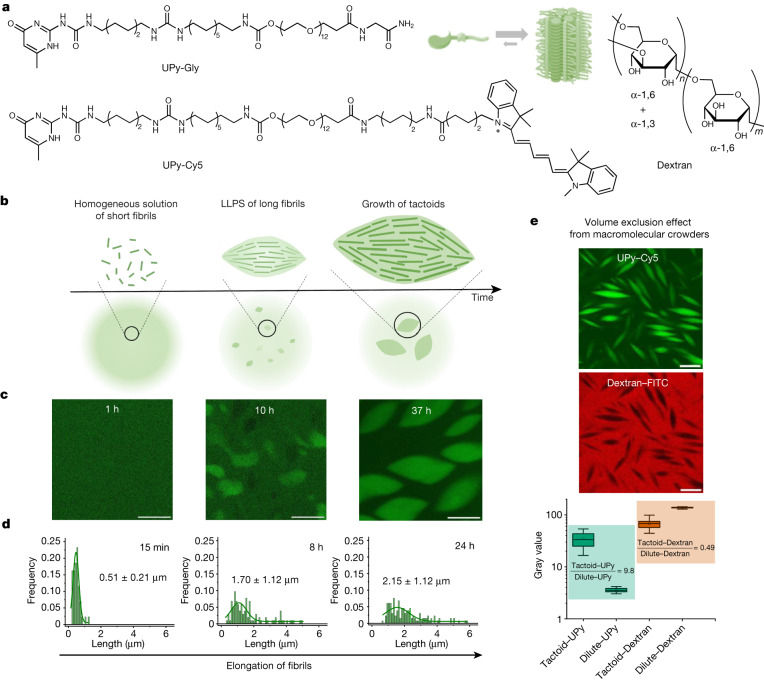
LLPS of supramolecular polymers through the entropy-driven pathway. **a**, Chemical structures of UPy-Gly, UPy-Cy5 and dextran (MW ≈ 500 kDa), and schematic representations of UPy-Gly and its supramolecular polymer. **b**, Schemes for the spontaneous phase separation of UPy-Gly supramolecular polymers into tactoids over time with the association and elongation of supramolecular polymer rods. **c**, The transition from homogeneous solution to liquid–liquid phase-separated solution followed by the growth of tactoids over time tracked by CLSM. The UPy-Gly (1 wt%, 8.4 mM) supramolecular polymers were labelled with 0.02 mol% of UPy-Cy5, PBS × 0.25, pH = 7.6. **d**, The length distributions of fibrils tracked by AFM show fibril growth and more heterogeneous populations over time. **e**, LLPS of UPy-Gly supramolecular polymers (top, labelled by UPy-Cy5) could be promoted by the volume exclusion effect of dextran (1.5 wt%, middle, labelled by 0.08 mol% of dextran-FITC). The bottom graph shows the partitioning of UPy-Gly and dextran in the two phases. Scale bars, 20 μm (**e**), 50 μm (**c**).

**Extended Data Fig. 1:**
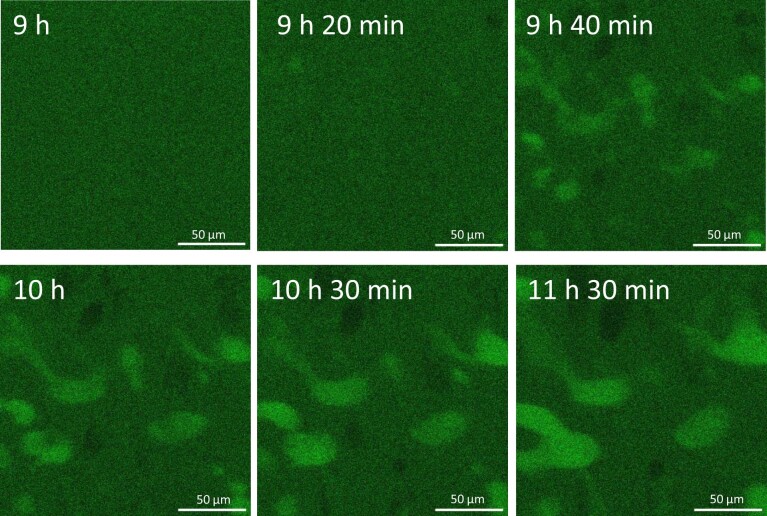
Phase separation process for the UPy-Gly solution with no dextran. Solution condition: UPy-Gly 1 wt% (8.4 mM), UPy-Cy5 1.8 µM, pH = 7.7, PBS × 0.25. The scale bar is 50 µm.

By studying the supramolecular polymerization of 1 wt% of ureidopyrimidinone glycine (UPy-Gly) in an aqueous solution, with a small fraction of the dye UPy-Cy5 (less than 0.1 mol%), we observed the overnight transition from a homogeneous solution to a heterogeneous LLPS solution with confocal laser scanning microscopy (CLSM) (Fig. 1a–c and Extended Data Fig. 1). This coexistence of concentrated and diluted liquid regions in the aqueous solution reminded us of macromolecular solutions undergoing LLPS. Even more striking was that the shape of the droplets was not spherical but spindle-like, reminiscent of the tactoids formed by biofilaments^19,21^. Inspired by the crowding effect, we introduced dextran as a macromolecular crowder into the UPy-Gly solution to tune both the supramolecular polymerization and phase-separation behaviour (Fig. 1e). The coupling of supramolecular polymerization and phase separation eventually led to dynamic, highly ordered, three-dimensional (3D) structures up to millimetre or even centimetre scale (Fig. 4 and Extended Data Fig. 6).

## Driving forces for LLPS

**Extended Data Fig. 2:**
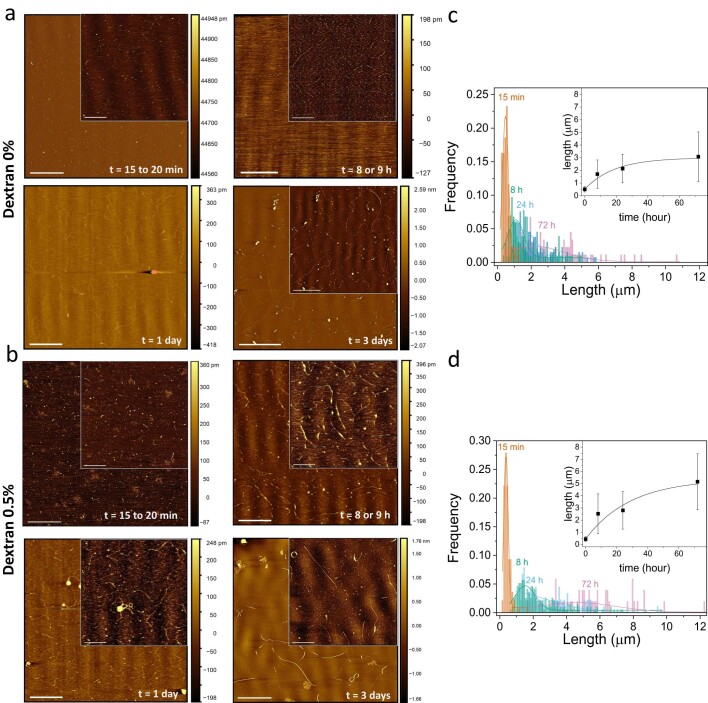
Length distributions of UPy-Gly fibrils from AFM measurements for t = 15 min, 8 h, 1 day and 3 days. **a**-**b**, Representative AFM images at t = 15 to 20 min, 8 or 9 h, 1 day and 3 days in the presence of 0 wt% (a), 0.5 wt% (b) of dextran. The scale bars for the insets and large pictures are 2 and 4 µm, respectively. **c**-**d**, Histograms of the fibril length distributions at different time for solutions with 0 wt% (c), 0.5 wt% (d) of dextran. The inset is a plot of the average length of the fibrils over time. UPy-Gly 1 wt%, pH = 7.6, PBS × 0.25. n = 43 to 195.

**Extended Data Fig. 3:**
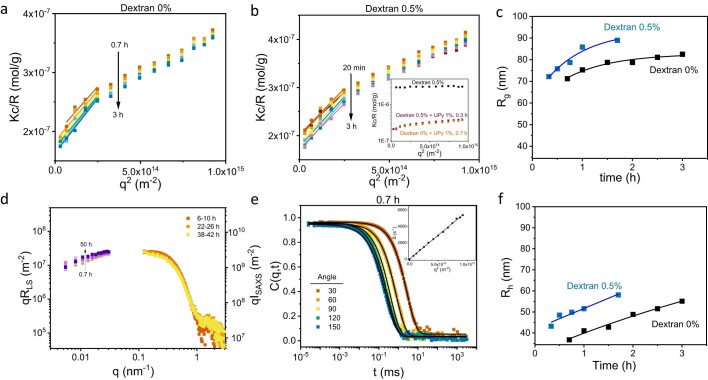
Tracking the growth of fibrils with light scattering. **a**-**b**, The reciprocal reduced intensity *Kc/R* in a “Zimm” representation Ornstein of 1 wt% of UPy-Gly solution with 0 wt% (a) and 0.5 wt% (b) of dextran at different time (arrows). The inset in b) shows the very small dextran contribution to the total light scattering intensity of the fibril solution. **c**, Radius of gyration, *R*_g_, obtained from the slope of the *Kc/R* in the linear Guinier regime in a) and b); *q* is the reciprocal probing length. **d**, Light and X-ray scattering intensity patterns with 0 wt% of dextran in a Holtzer representation to indicate the fibril contribution (at low *q*’s), the rod-like regime (*R* ~ *q*^−1^) at *q* ~ 0.03 nm^−1^ with a persistent length of ~ 100 nm and the local packing of fibrils (at *q* > 0.2 nm^−1^). **e**, Relaxation function *C(q,t)* of the concentration fluctuation in the dilute fibril regime with 0 wt% of dextran at different scattering angles (*q*’s). The single diffusion coefficient, *D*_f_, is indicated by the *q*^2^ dependent relaxation rate (inset). **f**, The hydrodynamic radius *R*_h_ with 0 wt% and 0.5 wt% of dextran at early time (dilute fibril regime). More details are given in the Supplementary Information.

We observed the tactoids by coincidence while studying a diluted solution of water-soluble supramolecular polymers that are a few days old. Previous studies showed that UPy dimerization and stacking of the units result in the formation of isolated, semirigid supramolecular polymers immediately after preparing the solution, as observed by cryogenic transmission electron microscopy (cryoTEM). This early observation of tactoids started a detailed study on the phase behaviour of these one-dimensional polymers. The change in structure and morphology over time of fresh solutions of UPy-Gly (1 wt%, pH = 7.6 ± 0.2, phosphate-buffered saline (PBS) × 0.25 with 0.02 mol% of UPy-Cy5) were first studied by atomic force microscopy (AFM) and CLSM. We observe that fibrils are formed, which grow significantly longer over time before tactoids appear (from 0.5 ± 0.2 μm to 1.7 ± 1.1 μm in 8 h; Fig. 1d and Extended Data Fig. 2a). The hydrodynamic radius, *R*_h_, obtained from dynamic light scattering (DLS) in the dilute fibril solution, increases with time. The *R*_h_ values along with the radius of gyration, *R*_g_, conform to semirigid chains with a persistent length of the order of 100 nm (Extended Data Fig. 3c,e,f and Supplementary equation (14))^36^. Even when LLPS occurs, the fibrils continue to grow (Fig. 1d and Extended Data Fig. 2). When we fragment the long fibrils in the aged, phase-separated solution into short fibrils (0.15 ± 0.05 μm) using dual asymmetric centrifugation, the solution becomes homogeneous again. In this case the phase separation reappears only 7 days later owing to the drastically reduced growth rate of aged fibrils (Supplementary Fig. 1). Thus, a critical length of supramolecular fibrils is required before macroscopic LLPS is observed.

**Extended Data Fig. 4:**
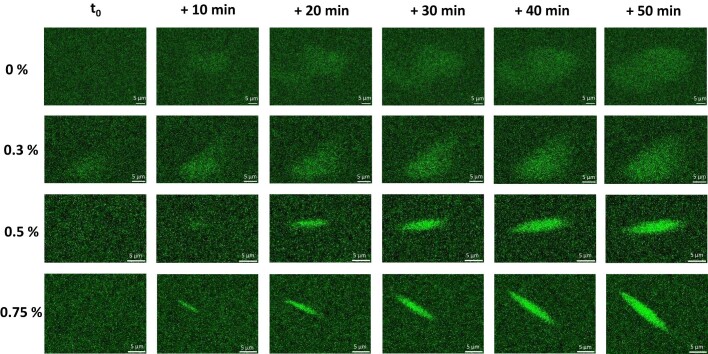
The growth process of tactoids with different concentrations of dextran from t_0_ to t_0_ + 50 min. t_0_ corresponds to the transition point in Fig. 2c. It’s dependent on the dextran concentration. Solution condition: UPy-Gly 1 wt%, UPy-Cy5 1.8 µM, pH = 7.6 or 7.7, PBS × 0.25, dextran 0 wt%, 0.3 wt%, 0.5 wt% and 0.75 wt%. The scale bar is 5 µm.

To understand the origin of the tactoids formed by LLPS of supramolecular polymers, we analysed the chemical and geometrical properties of the UPy–Gly polymers, as the free energy change during LLPS is controlled not only by the solvent–solute interactions, but also by the size and shape of the solutes^8,10,26^. The partially exposed hydrophobic groups and the amphiphilic nature of the end groups allow continuous elongation and relatively weak parallel association of the UPy-Gly polymers (Fig. 1a). At the same time, the negative zeta potential exerted by the ureidopyrimidinone functional group is modulated by pH and salt concentration to partially counteract these associative interactions (Supplementary Fig. 4). Geometrically speaking, the supramolecular polymers are rod-like, and their size and aspect ratio increase with time owing to the longitudinal supramolecular polymerizations. This suggests that the UPy–Gly polymers have weak repulsive interactions with water and the elongation of fibrils causes the transition of the solution from a stable to a metastable state, in which phase separation occurs by means of the nucleation-growth pathway instead of spinodal decomposition (Extended Data Figs. 1 and 4; for more discussions, see Supplementary Information). The spontaneous ordering within the tactoids should be a result of maximized translational entropy at the cost of rotational entropy of the high-aspect-ratio fibrils according to Onsager’s theory^26^. A study on the temperature effect shows that the phase separation is faster and results in smaller tactoids at higher temperature, suggesting that LLPS behaviour is entropically favourable (Supplementary Figs. 15 and 16).

## Crowding effect on LLPS

**Fig. 2:**
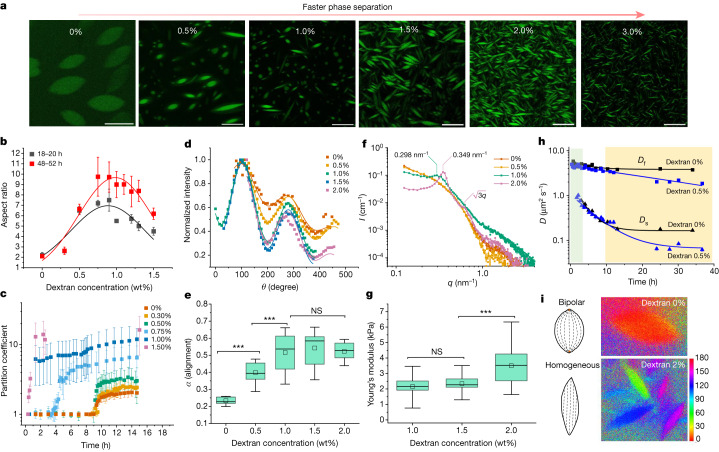
Controlling the LLPS of UPy-Gly supramolecular polymers with dextran concentration. **a**, Variations in the morphology and the number density of tactoids as a function of dextran concentration. The UPy-Gly (1 wt%, 8.4 mM) supramolecular polymers were labelled with UPy-Cy5. **b**, Change in the long axis to short axis ratio (aspect ratio) as a function of dextran concentration and time (*n* = 3–19). **c**, Change in the phase separation states and kinetics as a function of dextran concentration (*n* = 6–26) tracked by the fluorescence intensity of UPy-Cy5 using CLSM. **d**, Plot of the normalized fluorescence intensities as a function of the angle of the polarized excited beam for 2-day-old solutions with different dextran concentrations. Solid lines are the fits to the data as explained in the Supplementary Information. **e**, Plot of the fibril alignment factors for 2-day-old solutions with different dextran concentrations (*n* = 12–18). The UPy-Gly (1 wt%, 8.4 mM) supramolecular polymers were labelled with 1.8 μM of UPy-Cy5 (0.02 mol% of UPy–Gly). **f**, SAXS profiles showing the impact of dextran concentration on the packing of the fibrils. **g**, Change in the Young’s modulus of the 8-day-old tactoids at different dextran concentrations measured with AFM (*n* = 10–14). **h**, Fibril self-diffusion constant, *D*_s_, and cooperative diffusion constant, *D*_f_, after 3 or 4 h with 0 wt% (black) and 0.5 wt% (blue) of dextran, extracted from DLS. The areas define dilute (green), semidilute (white) and LLPS (yellow) with increasing time. **i**, POLCAM fluorescence images of tactoids in the presence of 0 wt% (top) and 2 wt% (bottom) of dextran. Both samples are 1 day old. The colour bar on the right represents the linear polarization angle (unit: degree) of the emission of UPy-Cy5 against an arbitrary 0° direction in the *x–y* plane. Scale bars, 50 µm. NS, not statistically significant; ***P < 0.01.

As the concentration of UPy-Gly is rather low (1 wt%), we introduced dextran (MW ≈ 500 kDa) as the macromolecular crowder into the UPy–Gly solution to accelerate the phase-separation process. Adding crowders leads to the synergistic acceleration of the fibril elongation, as well as to the formation of tactoids (Extended Data Figs. 2 and 3). Our initial focus was on the morphology of tactoids. We used UPy-Cy5 or Nile red to follow the process: Nile red would colour the hydrophobic domains and UPy-Cy5 is co-assembled into the fibrils. By increasing the concentration of dextran, the LLPS process becomes faster, and the tactoids formed are smaller in size, thinner in shape and, as a result, more dense and rigid (Fig. 2a). Quantification of the aspect ratio as a function of the dextran concentration (0–1.5 wt%) results in a bell-shaped curve that reaches a maximum at approximately 0.9 wt% of dextran (Fig. 2b). At 3 wt% of dextran and above, the tactoids look more like long filaments/bundles (for discussions, see Supplementary Information). Notably, aspect ratios of tactoids at 0.75–1.5 wt% of dextran increased by 30–70% from time *t* = 19 ± 1 h to 50 ± 2 h. For tactoids formed by amyloid fibrils, by contrast, the aspect ratio was reported to decrease over time because of the increasing volume of droplets. In the meantime, the aspect ratio was increased when the initial amyloid fibril length was longer^37^. Therefore, we propose that the increase in the aspect ratio of UPy-Gly tactoids over time, despite the increase of volume, can be attributed to the further elongation of fibrils as the result of supramolecular polymerizations. AFM experiments showed that the fibrils grew in length by five to ten times in 3 days and that the fibrils with 0.5 wt% of dextran were 30–70% longer than those without dextran (Extended Data Fig. 2). The hypothesis is further supported by the increase of *R*_g_ and *R*_h_, extracted from static light scattering (SLS) and DLS, in the beginning, and the decrease of fibril self-diffusion constant, *D*_s_, over a longer period (Fig. 2h and Extended Data Fig. 3). The strong decrease of *D*_s_ up to about 10 h is due to the increase of both fibril length and solution viscosity.

By tracking the concentration ratios of UPy-Cy5 inside and outside the droplets (the partition coefficient), we could follow the phase-separation process in real time (Fig. 2c). The incubation period was significantly shortened with increased dextran concentrations, which is likely to be the result of faster supramolecular polymerizations, shorter critical fibril length and/or accelerated nucleation of LLPS. The elevated plateaus of the concentration ratios suggest stronger phase separations with more crowders. The transition to the phase-separated state can also be detected by the slow diffusion of tactoids in the dilute phase, *D*_us_, using DLS (Supplementary Fig. 9). In conclusion, increasing crowder concentration accelerates phase separation; this separation increases the concentration of supramolecular polymers in the concentrated phases, which in turn promotes fibril elongation, a kind of autocatalytic effect. This conclusion also makes the question as to whether LLPS induces protein associations and/or whether associative interactions between biomacromolecules are required for LLPS less relevant,as both occur at the same time.

**Fig. 3:**
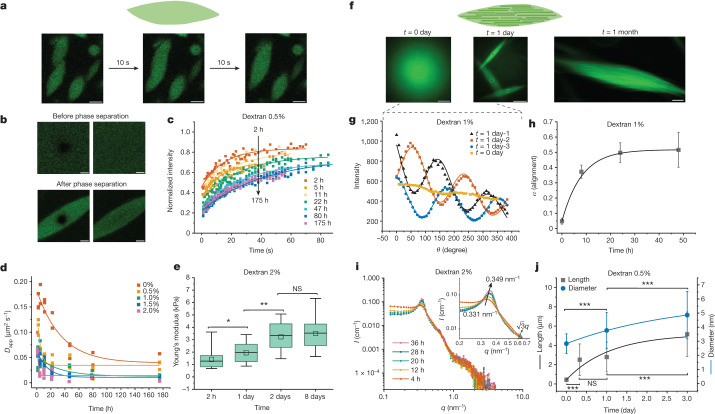
Time evolution of the fluidity and internal ordering of the tactoids and geometry change of supramolecular polymers. **a**, Spontaneous fusion of two tactoids in 20 s. The images were taken for the solution with 1 wt% (8.4 mM) of UPy-Gly, 2 wt% (0.04 mM) of dextran and 1.8 μM of UPy-Cy5, at pH = 7.6, PBS × 0.25 at *t* = 1 h. This is the default solution condition if not otherwise specified. **b**, Typical images for the FRAP experiment representing that the solutions (0.5 wt% of dextran) before and after phase separation were bleached (left) and reached a high percentage of recovery afterwards (right). **c**, The FRAP curves for the solution with 0.5 wt% of dextran at different ageing times. **d**, Plot of the diffusion constants extracted from the recovery curves at different ageing times for solutions with different dextran concentrations (0 wt%, 0.5 wt%, 1 wt%, 1.5 wt% and 2 wt%). **e**, The change of the Young’s modulus of the tactoids over time (2 wt% of dextran, two-sample *t-*tests were conducted, *n* = 10–14; the empty square in the box plot represents the mean). **f**, Representative images of the solution (0 wt% of dextran, *t* = 0 day) or tactoids (1 wt% of dextran, *t* = 1 day and 1 month) at different times. **g**, Representative curves of the fluorescence intensities in the circular areas of samples indicated in **f** at *t* = 0 and 1 day under linearly polarized excitation with polarization angle *θ*. **h**, The time evolution of the extent of fibril alignment extracted from the polarized fluorescence microscopy curves in Fig. 3g and Supplementary Fig. 12 (1 wt% of dextran, *n* = 5–14). **i**, SAXS profiles showing the regular distance between fibrils and more compact packing over time (2 wt% of dextran). **j**, Diameters and lengths of UPy-Gly fibrils from the cryoTEM and AFM images showing the increase of fibril width and length over time (0.5 wt% of dextran) (*n* = 51–194). Scale bars, 5 µm (**a**), 10 µm (**b**,**f**). *P ≤ 0.1, **P < 0.05, ***P < 0.01.

To understand and quantify the internal order of the tactoids, we conducted polarized fluorescence microscopy experiments. The fluorescence intensities of the tactoids were shown to be strongly dependent on the angle of the linearly polarized excitation beam with a period of 180°, which is typical of axially aligned fibrils (Fig. 2d and Supplementary Fig. 8). In the normalized intensity plot, the amplitude of the sinusoidal curves increases with the dextran concentration (Fig. 2d). The extent of alignment obtained by fitting the sinusoidal curves demonstrates that the fibrils are better aligned with the increase of dextran concentrations in a narrow range (Fig. 2e and Supplementary Fig. 8), in full agreement with the increased aspect ratio of the tactoids. To cross-check, we also used small-angle X-ray scattering (SAXS) to characterize the UPy-Gly solution. It showed the regular packing and enhanced order of individual fibrils with sharp peaks and increased peak intensities at higher dextran concentrations. The drifted peak position from 0.30 nm^−1^ to 0.35 nm^−1^ corresponds to an interfibril distance change from 21 nm to 18 nm, confirming more compact packing at a higher dextran concentration (Fig. 2f). This finding implies an increased internal viscosity, as also inferred from *D*_s_ in Fig. 2h. The peak near 0.62 nm^−1^ indicates hexagonal packing of the fibrils. The in situ AFM force measurements of the 8-day-old samples showed that the Young’s modulus of tactoids increased with the dextran concentration from 2.2 ± 0.8 kPa (1 wt% of dextran) to 3.5 ± 1.3 kPa (2 wt% of dextran), which may be a result of the more compact distributions of fibrils (Fig. 2g and Supplementary Fig. 5). The instant molecular orientation microscopy based on a polarization-sensitive camera (POLCAM) showed the liquid crystalline state of the tactoids (Fig. 2i)^38^. At 0 wt% of dextran, the tactoids are bipolar, whereby fibrils on the edge are tangentially anchored. In the presence of dextran (0.5–2 wt%), most tactoids transition to the more ordered ‘homogeneous’ configuration in which most fibrils are aligned with the long axis of the tactoids (Figs. 2i and 3f and Supplementary Fig. 10)^39^.

Besides, the crowding effect can be achieved by other macromolecules. The negatively charged alginate also promotes LLPS of UPy-Gly supramolecular polymers. Tactoids with similar morphology are formed with even lower concentrations (less than 1 wt%) of alginate because the negative charge in alginate can increase its excluded volume through both intramolecular and intermolecular repulsive interactions (Supplementary Fig. 3).

## Time evolution of LLPS

To confirm the liquid nature of the tactoids formed by the phase-separated UPy-Gly supramolecular polymers, we examined the tactoids with CLSM and AFM using different amounts of dextran. The fresh tactoids, using 2 wt% of dextran, fused together in 20 s on contact, demonstrating the fluid nature of the tactoids (1 wt% or 8.4 mM of UPy-Gly, *t* = 1 h; Fig. 3a). High percentages of fluorescence recovery within 1 min after photobleaching (FRAP) of the UPy-Cy5 were observed but the percentages of recovery decreased over a few days (Fig. 3b,c). The apparent diffusion constants (*D*_app_, Fig. 3d and Supplementary Fig. 11) obtained by fitting the recovery curves, are on the same order as the self-diffusion constants of fibrils (*D*_s_) obtained from DLS (Fig. 2h), indicating that the fluorescence recovery should have been realized by the diffusion of fibrils. *D*_app_ showed exponential decay over time, the rate of which increases with the dextran concentration. This could be due to accelerated fibril elongation with more crowders and the increase of the internal viscosity of tactoids (Fig. 3d and Extended Data Fig. 3). The in situ AFM force curve measurements showed continuously increasing Young’s modulus in 2 days (from 1.4 ± 0.5 kPa to 3.2 ± 1.0 kPa, 2 wt% of dextran), which is comparable to the mechanical strength of the cytoplasm (Fig. 3e and Supplementary Fig. 5)^40^.

To understand the change in the internal order of the tactoids, we tracked the UPy-Gly solution containing 1 wt% of dextran over time. The fluorescence intensity of the UPy-Cy5 from the homogeneous solution was barely responsive to the angle of the linearly polarized excitation beam other than some slight decay due to photobleaching (Fig. 3f,g). However, on phase separation, the fluorescence from the tactoids started to depend on excitation polarization with a period of 180°, indicating increasing internal alignment (Fig. 3f,g). By fitting the sinusoidal curves, a ten-fold increase in the extent of alignment within the tactoids was shown over 2 days (Fig. 3h and Supplementary Fig. 12).

To investigate the origin of changes in the liquid state and the internal order of the tactoids, we zoomed in on the individual fibrils. With cryoTEM and AFM, we observed that the fibrils became both thicker (from 2.8 ± 0.7 nm to 4.9 ± 1.7 nm, 0.5 wt% of dextran) and longer (from 0.4 ± 0.2 µm to 5.2 ± 2.3 µm, 0.5 wt% of dextran) in 3 days, revealing continuous supramolecular polymerizations coupled with LLPS (Fig. 3j, Extended Data Fig. 2 and Supplementary Fig. 2). An extreme case of the extended, bundled structures can be observed in the 1-month-old tactoids with CLSM (1 wt% of dextran; Fig. 3f and Supplementary Fig. 13). The increase in peak intensities and change in peak positions (from 0.33 nm^−1^ to 0.35 nm^−1^) shown by SAXS further consolidated the formation of more regularly and compactly distributed fibrils over time (2 wt% of dextran; Fig. 3i). The change in fibril dimensions and the interfibril spacing within the tactoids could explain the decreased diffusion rate and more rigid nature over time. The improved alignment could be attributed to the entropic effect after the elongation of fibrils.

## The effect of substrate–liquid interfaces

**Fig. 4:**
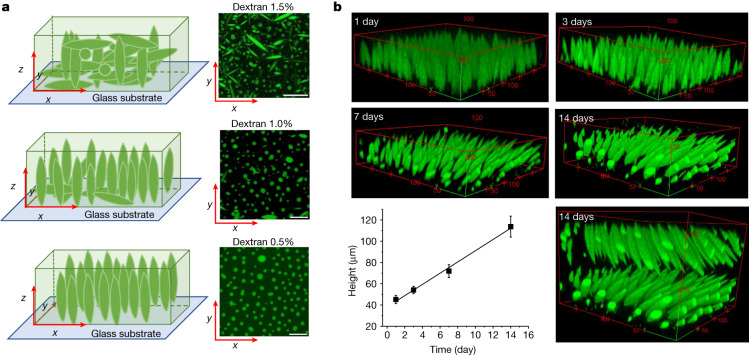
Interface directed vertical alignment of tactoids. **a**, The 3D distribution of tactoids at the water–glass substrate interface as a function of dextran concentration. Left, 3D schematic representations. Right, cross-section images (*t* = 14–19 h). **b**, 3D projections of the vertically aligned tactoids at *t* = 1, 3, 7 and 14 days (0.5 wt% of dextran) and the two-layer distribution of vertically aligned tactoids in the presence of two water–glass interfaces at *t* = 14 days. The bottom left is the plot of the height of the tactoids as a function of time (*n* = 10–36). The unit of the *xyz* axis is micrometres. The UPy-Gly (1 wt%, 8.4 mM) supramolecular polymers were labelled with 1.8 μM of UPy-Cy5 (0.02 mol% of UPy-Gly). All experiments have been replicated at least two to three times. Scale bars, 50 µm.

**Extended Data Fig. 5:**
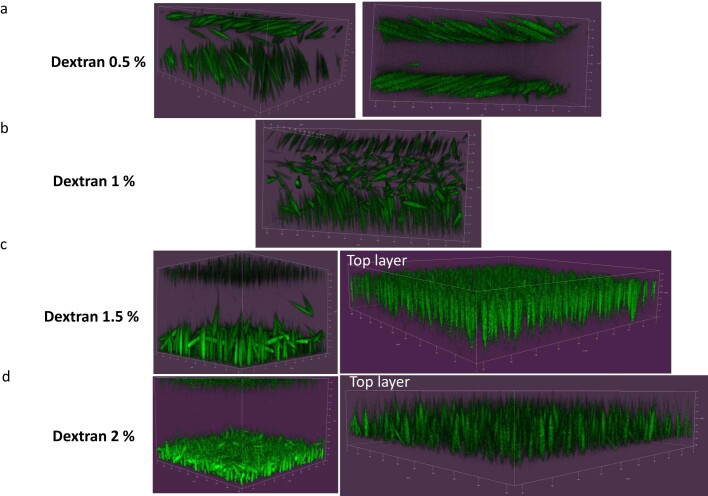
3D images of tactoids with 0.5 wt% of UPy-Gly and different concentrations of dextran. **a**-**d**, Solution condition: UPy-Gly 0.5 wt%, UPy-Cy5 0.9 µM, dextran 0.5 wt% (a), 1 wt% (b), 1.5 wt% (c) and 2 wt% (d), pH = 7.6, PBS × 0.25, t = 12 days. The samples were incubated in a 120 μm thick chamber with one piece of glass on the top and one at the bottom. The unit of the xyz axis is μm.

**Extended Data Fig. 6:**
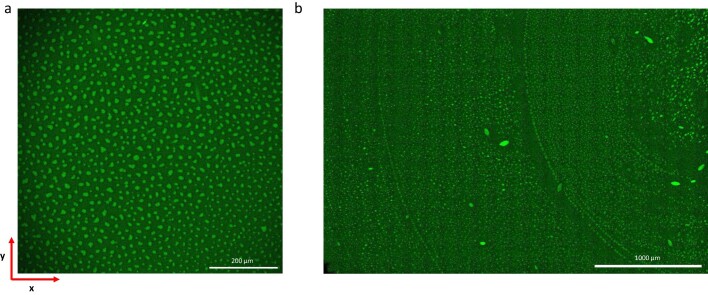
Vertical tactoids visualized from the x-y plane with confocal microscope. The non-spherical shape was caused by the merging with nearby tactoids on the substrate. Aging can slow down the fusion of tactoids. In addition, the cross sections are also less regular close to the substrate, which may be due to the anchoring effect of the substrate that has slowed down the fusion of the bottom parts of tactoids. Solution condition: UPy-Gly 1 wt%, UPy-Cy5 1.8 µM, pH = 7.6, PBS × 0.25, dextran 0.5 wt% (a, t = 25 h) and 0.75 wt% (b, t = 27 h). The scale bar is 200 µm for (a) and 1000 µm for (b).

**Extended Data Fig. 7:**
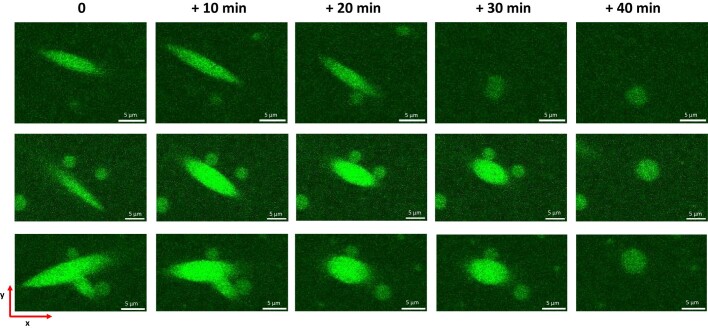
Slow flipping of flat tactoids and fast fusion with one or more vertical tactoids. Each column is separated by 10 min. Each row is one independent spot. Solution condition: UPy-Gly 1 wt%, Nile Red 0.5 µM, dextran 1 wt%, pH = 7.7, PBS × 0.25, t = 3 to 4 h. The scale bar is 5 µm.

**Extended Data Fig. 8:**
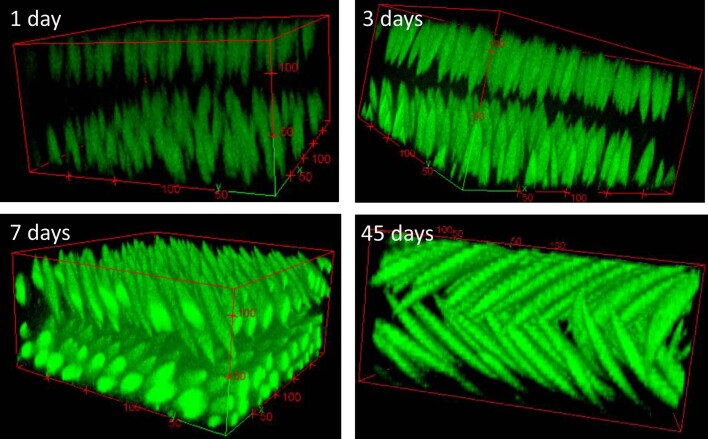
3D images from confocal z scanning for double layered, vertical tactoids with 0.5 wt% of dextran at different time. Time: 1 day, 3 days, 7 days and 45 days. Solution condition: UPy-Gly 1 wt%, UPy-Cy5 1.8 µM, dextran 0.5 wt%, pH = 7.6, PBS × 0.25. The sample was incubated in a 120 μm thick chamber with one piece of glass on the top and one at the bottom. The unit of the xyz axis is μm.

LLPS of UPy-Gly supramolecular polymers at the substrate–liquid interface occurred several minutes to several hours/days earlier (in a narrowly confined space) than in the bulk solution containing a dextran concentration of 0.5 wt% or lower. This is consistent with the report that the substrate can nucleate new liquid phases or states^41–43^. With 1.5 wt% or higher concentrations of dextran, the nucleation effect of the interface was less pronounced, and the phase separation happened almost simultaneously at the substrate–liquid interface and in the solution. More strikingly, highly ordered arrays of vertical tactoids were obtained at the substrate–liquid interface, compared with the less oriented tactoids in the solution (0.5–2.0 wt% of dextran; Fig. 4, Extended Data Fig. 5 and Supplementary Video 1). Large areas (millimetre to centimetre in diameter) of the substrate were found to be covered with vertical tactoids that look circular on the *x*–*y* plane (0.5 wt% of dextran; Extended Data Fig. 6). The tactoids rooting on the substrates could slowly tilt/diffuse and merge with their neighbours, generating larger tactoids. The height of the tactoids was found to increase almost linearly in 14 days (0.5 wt% of dextran; Figs. 3d and 4b). On the other hand, real-time tracking of areas, tens of micrometres above the substrate, revealed that some flat tactoids in the solution were able to slowly flip and quickly merge with one or several vertical tactoids, increasing the overall order in the *z* direction of the bulk solution (1 wt% of dextran; Supplementary Video 2 and Extended Data Fig. 7). Note that the fusion of tactoids is fast in the beginning and gradually slows down during ageing, which can give rise to trapped fusion states in transition (Supplementary Fig. 19). When the solution was confined between two substrates, two layers of vertical tactoids could be formed, both nucleated from their own surfaces (0.5 wt% of dextran; Fig. 4b and Extended Data Fig. 8). Moreover, the density of the tactoids seemed to increase with dextran concentration. At relatively low concentrations of dextran (less than or equal to 1 wt%), the tactoids generated from the solution were able to float, generating multilayered structures (Supplementary Video 1 and Extended Data Fig. 5b). At higher concentrations of dextran (1.5–2.0 wt%), the tactoids formed in the bulk solution would sink to the bottom because of gravity, leaving an almost perfectly aligned top layer, an empty middle layer and a less ordered bottom layer mixed with vertical tactoids and flat ones falling from the bulk solution (Fig. 4a, Supplementary Fig. 14 and Extended Data Fig. 5c,d). Several reasons can be given for the vertical alignment of tactoids on the substrates, including the nucleation of the metastable solution at the substrate–liquid interface overruling fibril–substrate interactions and sample flow while mounting the glass substrate^44–48^.

## The effect of liquid–liquid interfaces

**Fig. 5:**
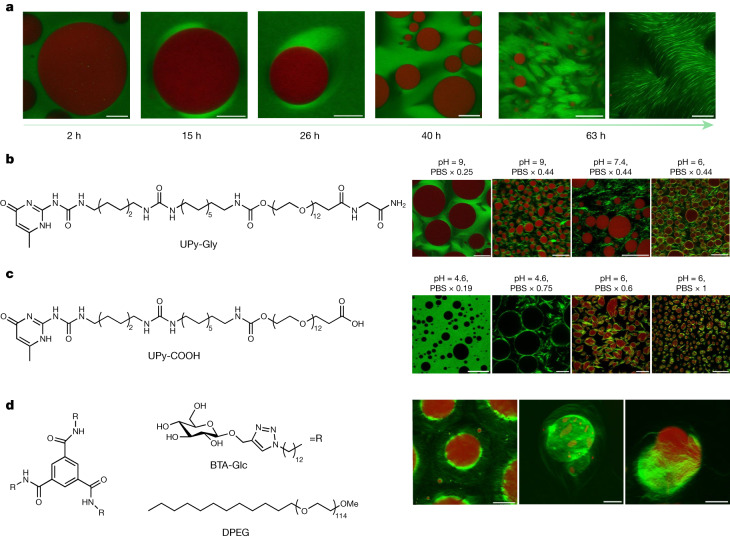
The phase-separation of supramolecular polymers at the liquid–liquid interface. **a**, The LLPS of UPy-Gly supramolecular polymers was accelerated at the PEG–dextran interface. Solution composition: 1 wt% (8.4 mM) of UPy–Gly, 5 wt% of dextran and 5 wt% of PEG; pH = 9, PBS × 0.19. Red: dextran-FITC, green: Nile red or UPy–Cy5. **b**,**c**, Stabilization and deformation of the dextran droplets (red or dark) after the phase separation and ageing of the UPy-Gly (**b**) and UPy-COOH (**c**) supramolecular polymers under different pH and PBS concentrations, *t* = 1 day. **d**, Stabilization of the dextran droplets after the phase separation and ageing of the BTA-Glc supramolecular polymers. The hairy ball-like BTA-Glc cage (Nile red, green) could encapsulate the dextran droplets inside and release the droplets under pressure. Solution composition: 0.22 wt% (1.5 mM) of BTA-Glc, 0.18 wt% (0.34 mM) of DPEG, 8 wt% of PEG and 2 wt% of dextran, *t* = 3 or 4 days. All experiments have been replicated at least two to three times. Scale bars, (left to right) 50, 10, 10, 50, 200 and 50 µm (**a**), 50, 20, 50 and 20 µm (**b**), 50, 20, 20 and 50 µm (**c**), 50, 20 and 20 µm (**d**).

Because biopolymer fibrils stabilize droplets in cells, it can be intuitively assumed that the accelerated LLPS, presented above, could also be extended to these liquid–liquid interfaces to preferentially form these ordered structures around droplets^49^. To test this idea, we constructed the liquid–liquid interface with the well-known polyethylene glycol (PEG)–dextran aqueous two-phase system, where dextran droplets can be generated in the continuous phase of PEG (Supplementary Fig. 20)^50,51^. On mixing the supramolecular polymers of UPy-Gly with the PEG–dextran solution, the fibrils partitioned preferentially to the PEG phase as it is chemically more like PEG (Fig. 5a and Extended Data Fig. 10b). When the dextran droplets are removed by centrifugation, the supramolecular polymers phase-separate into tactoids (Supplementary Fig. 23). In the presence of dextran droplets, crown-shaped UPy-Gly supramolecular polymer condensates (tactoids distorted by the circular interface) are first initiated at the droplet interface, precedent to LLPS in the bulk solution of the PEG-rich phase (Fig. 5a and Supplementary Video 3). Subsequently, gradually aligned bundles formed within the supramolecular polymer condensates, eventually generating a network that stabilized the droplets (Fig. 5a,b). pH and salt concentrations can change the bundling state of UPy-Gly supramolecular polymers and the morphology of tactoids probably because of their influence on the zeta potential of the supramolecular polymers (Supplementary Figs. 1, 17, 18, 21 and 22). As a result, they can also influence the supramolecular polymer networks. By varying pH and salt concentrations, continuous or discrete supramolecular polymer networks could be generated that can either support or deform the dextran droplets (Fig. 5b; for more discussions, see Supplementary Information).

**Extended Data Fig. 9:**
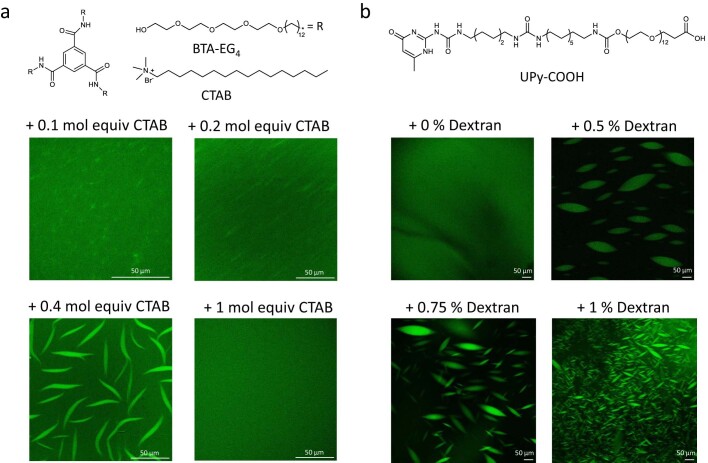
Generalization of LLPS behavior in other supramolecular polymer systems. **a**, 4 mg/mL of BTA-EG_4_ mixed with 0.1, 0.2, 0.4 and 1 mol equivalent of CTAB, t = 6 days. BTA-EG_4_ was labelled with BTA-Cy5. **b**, 1 wt% of UPy-COOH mixed with 0 wt%, 0.5 wt%, 0.75 wt% and 1 wt% of dextran at pH = 7.2 and PBS × 0.25, t = 12 h. UPy-COOH was labelled with 0.5 μM of Nile Red. The scale bar is 50 µm.

**Extended Data Fig. 10:**
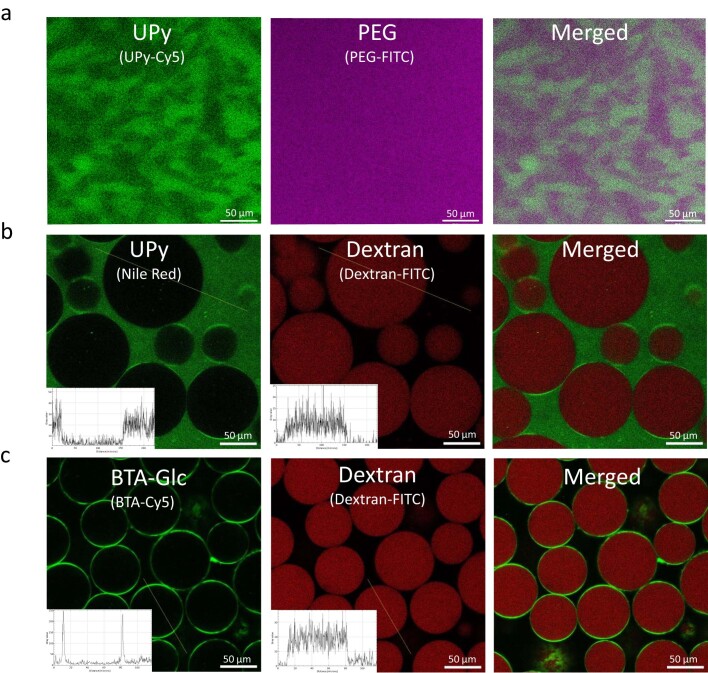
Partition of UPy-Gly and BTA-Glc in the ATPS of PEG and Dextran. **a**, 1 wt% of UPy-Gly with 7 wt% of PEG, pH = 9, PBS × 0.25, t = 1 day. UPy-Gly was labelled with 0.02 mol% of UPy-Cy5. PEG was labelled with 0.1 mol% of PEG-FITC. **b**, 1 wt% of UPy-Gly in 5 wt% of PEG and 5 wt% of dextran, pH = 9, PBS × 0.25, t = 3 h. UPy-Gly was labelled with 0.5 µM of Nile Red. Dextran was labelled with 0.08 mol% of dextran-FITC. The insets are the intensity profiles of Nile Red (UPy-Gly) and dextran-FITC along the yellow lines, respectively. **c**, 2.2 mg/mL of BTA-Glc and 1.8 mg/mL of DPEG with 8 wt% of PEG and 2 wt% of dextran, t = 3 h. BTA-Glc was labelled with 2 µM of BTA-Cy5. Dextran was labelled with 0.08 mol% of dextran-FITC. The insets are the intensity profiles of BTA-Cy5 and dextran-FITC along the yellow lines, respectively. The scale bar is 50 µm.

According to the Flory–Huggins theory, LLPS should be a general phenomenon for different supramolecular polymer systems given appropriate solvent conditions, concentrations and chain length. To verify this hypothesis, we tested UPy-COOH and benzene-1,3,5-tricarboxamide (BTA)-EG_4_ in the aqueous solution and found that both could form into tactoids (Extended Data Fig. 9; see also discussions in Supplementary Information). To further validate this idea, we loaded both molecules to the PEG–dextran solution. The behaviour of UPy-COOH was generally similar to that of UPy-Gly, except for its response to pH and salt concentrations (Fig. 5c and Supplementary Figs. 4b and 24). BTA-EG_4_ failed to stabilize the dextran droplets as it aggregated strongly in the PEG phase and could not attach to the dextran droplets. BTA-Glc, on the other hand, could interact with dextran through the glucose functional group and tended to condense at the interface of the droplets (Fig. 5d and Extended Data Fig. 10c). The surfactant, dodecyl PEG (DPEG), was used to break down the heavily entangled network of BTA-Glc to enhance its solubility and mobility in the PEG phase. The condensed supramolecular polymers of BTA-Glc then transformed into bundles and extended to the PEG phase, forming into hairy ball-like structures with droplets encapsulated inside (Supplementary Video 4 and Fig. 5d). By pushing the droplets with the weight of the coverslip, the droplets were released, leaving most parts of the BTA-Glc cages intact (Fig. 5d).

## Discussion

We have developed a new way to produce highly ordered liquid tactoids with micrometre dimensions. The formation is based on a spontaneous LLPS behaviour of high-aspect-ratio fibrils, which is supposed to be a general phenomenon for various rod-like supramolecular polymers based on phase-separation theory. The process starts with the growth of supramolecular polymers and fibril elongation, followed by an entropy-driven phase separation to one ordered aqueous phase in a continuous aqueous solution with a low concentration of fibrils. The exact nature of the highly structured aqueous phase is intriguing and requires more investigation. Over time, the tactoids become more rigid. The rigidifying rate and extent increase with the concentration of crowders. Ordered arrays of structures over multi-length scales could be achieved in liquids by controlling LLPS and supramolecular polymerizations with solution conditions (pH and salt concentration), crowding effects and heterogeneous nucleation at the interface with the substrate. In addition, the dynamic behaviour caused by the liquidity of the new phase and the non-covalent nature of the intermolecular interactions makes it responsive to the actively evolving chemical system and the interactive environment. With tunable kinetics, dynamics, internal order, morphology and mechanics and many possibilities to combine different functional groups, we propose LLPS of supramolecular polymers as a general way to produce biomaterials that are applicable in the biological, medical and pharmaceutical fields by serving as the active interface with cells and tissues. Besides, the notable observation of LLPS of supramolecular polymers qualifies them as a new and amplified analogue to the low molecular weight liquid crystals and gives important insights into understanding the liquid crystal self-assembly. Moreover, the simplicity of the synthetic systems together with a high chemical diversity to modify the behaviour of LLPS make it an attractive model to explain many of the intriguing challenges of biological equivalents. The introduction of chiral and stimuli-responsive supramolecular polymers is notable and is part of further detailed studies.

## Methods

### Materials

All chemicals and reagents were purchased from commercial sources at the highest purity available and used as received unless otherwise stated. UPy-Gly, BTA-EG_4_ and BTA-Glc were ordered from SymoChem. mPEG-O-C12 (DPEG, PEG MW: 5,000), PEG-fluorescein isothiocyanate (FITC) (MW: 10,000) and dextran-FITC (MW: 550,000) were obtained from Creative PEGWorks. PEG (MW: 8,000), cetrimonium bromide (CTAB) and alginate and sodium salt from brown algae were purchased from Sigma-Aldrich. Dextran 500 was ordered from Pharmacosmos (pharmaceutical quality). Water was purified on an EMD Millipore Milli-Q Integral Water Purification system. UPy–COOH was synthesized following previous methods^52,53^. The synthesis of BTA-Cy5 is also reported in our previous work^54^. The synthesis route for UPy-Cy5 can be found in the Supplementary Information.

### Sample preparation

#### UPy-Gly or UPy-COOH stock solution

The stock solution was always freshly made. To prepare 4 wt% of stock solution, either UPy-Gly or UPy-COOH solid was dissolved in the PBS buffer and a calculated amount of 1 M NaOH, to make the final pH higher than 11. The solution was then heated at 75 °C for 15 min. Subsequently, a calculated amount of 600 μM of UPy-Cy5 or 1 mM of Nile red in methanol was added and mixed with the stock solution to reach a final concentration of 7.2 µM (UPy–-Cy5) or 2 µM (Nile red). Finally, 1 M HCl was added to adjust the pH to the targeted pH.

#### UPy-Gly or UPy-COOH solution with dextran

The freshly made UPy-Gly or UPy-COOH stock solution was immediately added into the prediluted PBS solution and mixed. Then 10 or 20 wt% of dextran solution (with 0.08 mol% of dextran-FITC) was added to reach to the required concentration. The mixture was vortexed for 20–30 s.

#### UPy-Gly or UPy-COOH solution with PEG and dextran

The freshly made UPy-Gly or UPy-COOH stock solution was immediately added into the premixed PBS and PEG solution and mixed. To achieve the required concentration, 10 or 20 wt% of dextran solution (with 0.08 mol% of dextran-FITC) was then added. The mixture was vortexed for 20–30 s.

#### BTA-EG_4_ solution with CTAB

A total of 4 mg ml^−1^ of BTA-EG_4_ stock solution was freshly made by mixing BTA-EG_4_ solid with 0.1 mol equiv. of CTAB and water; it was stirred and heated at 80 °C for 15 min. After heating, 500 µM of BTA-Cy5 in methanol was added to get a final concentration of about 1 µM. A small volume of the concentrated CTAB solution (50 mg ml^−1^) was added to adjust the BTA-EG_4_/CTAB ratio later.

#### BTA-Glc solution with PEG and dextran

A total of 4 mg ml^−1^ of BTA-Glc stock solution was prepared by dissolving BTA-Glc solid in water; it was stirred and heated at 75 °C for 15 min. After heating, 500 µM of BTA-Cy5 in methanol was added to get a final concentration of 1 or 2 µM. The stock solution was then mixed with the heated dPEG solution. Next, the preheated 20 wt% of PEG was added before adding 20 wt% of dextran solution (with 0.08 mol% of dextran-FITC). The volumes of the added solutions were adjusted to arrive at the designed concentrations. The solution was shaken or vortexed after each step. All solutions except for the UPy-Gly or UPy-COOH stock solution were either incubated in the Eppendorf tube or transferred to the imaging chambers immediately after preparation.

### Zeta potential measurements

Zeta potentials were measured on a Malvern instrument Zetasizer, model Nano ZSP. Zetasizer software was used to analyse and process the zeta potential data. For the preparation of UPy-COOH and UPy-Gly samples, the solid samples were dissolved and annealed in basic 0.84 × PBS (110 mM NaOH) at a concentration of 4.4 wt% for 15 min at 75 °C. After dissolving, the samples were diluted with MiliQ and 1 × PBS and adjusted to the final pH varying from 4.6 to 9 with 1 M HCl, resulting in 0.4 mM of UPy-Gly or UPy-COOH with different PBS concentrations. A DTS1070 cuvette was used for measuring the zeta potential. The measurement duration time was automated, and automatic attenuation and voltage was selected. The samples were measured in triplo at RT with a 30 s equilibration time.

### Dual asymmetric centrifugation

To break down UPy-Gly fibrils, dual asymmetric centrifugation was performed using a Hettich ZentriMix 380 R equipped with a ZentriMix rotor. Samples were added to 0.2 ml Eppendorf tubes and centrifuged for 60 min with a rotational speed of 2,500 rpm. The samples were subjected only to shear forces generated by the dual rotor set-up with no milling beads added.

### Confocal laser scanning microscopy

#### Sample preparation method 1

A total of 8–9 µl of the fresh sample was loaded into a 120-µm-thick chamber built with two pieces of #1.5 cover glass with an imaging spacer (Grace Bio-Labs SecureSeal imaging spacer, 8 wells, diameter × thickness: 9 mm × 0.12 mm) in the middle.

#### Sample preparation method 2

A total of 45–48 µl of the fresh sample was loaded into an 800-µm-thick chamber (Grace Bio-Labs SecureSeal hybridization chambers, 8 wells, diameter × depth: 9 mm × 0.8 mm, port diameter: 1.5 mm) with the #1.5 cover glass at the bottom. The open ports were sealed after sample loading.

#### Sample preparation method 3

About 100 µl of the fresh sample was loaded into each well of the ibidi μ-slide (18 wells, no. 1.5H glass bottom, well size: 5.7 × 6.1 × 6.8 mm^3^) and covered with the lid.

#### Sample preparation method 4

About 5 µl of the sample was loaded onto the glass holder and covered with the #1.5 cover glass. The surroundings of the cover glass were sealed with nail polish to reduce evaporation-induced drifting or flowing.

The fluorescent images and videos were acquired with the Leica TCS SP8 microscope in the confocal mode with 40×/0.9 (dry), 63×/1.2 (water immersion) and 63×/1.3 (oil immersion) objectives at a resolution of 512 × 512, 1,024 × 1,024 or 2,048 × 2,048 pixels. The lasers used were 488 nm (for FITC), 552 nm (for Nile red) and 638 nm (for Cy5). The videos were taken with a time gap of 10 min. The *z*-stack images were collected with a gap of 0.5, 1.0 or 2.0 µm.

The bright field images were collected with the Leica TCS SP8 microscope in the BF mode.

The image analysis was conducted with ImageJ or the Leica microscope built-in software (only for some *z*-stack images).

The tracking of the phase-separation states (concentration ratios of UPy-Cy5 inside and outside the droplets) is realized by time-lapsed videos of CLSM. The tempo-spatially resolved change of the fluorescence intensities can be extracted from each frame of the video. To avoid a bleaching effect, we set a 10 min gap between each frame. Several samples were tracked simultaneously using the ‘mark & find’ function.

### Fluorescence recovery after photobleaching

The FRAP was conducted on the Leica TCS SP8 microscope in the confocal mode with a 63×/1.2 (water immersion) numerical aperture objective at a resolution of 512 × 512 pixels. The samples were dyed with 1.8 µM of UPy-Cy5. One to three images were taken before bleaching with the imaging power of 0.5–1.0%. Subsequently, selected circular areas with diameters ranging from 0.6 to 7.0 µm were bleached for 10 cycles (0.3–0.7 s per cycle) at 50% of power with the 638 nm laser. The following images were captured with 0.5–1.0% of power every 0.3–5.0 s. The whole process was automated with the built-in software. Three or more measurements were conducted for each sample. The fluorescence intensities of the regions of interest (ROIs) were extracted by ImageJ and normalized with the intensities of the reference area following the equation below.

1 $$i\left(t\right)=\frac{I(t)/R(t)}{I(0)/R(0)}$$

Where *i*(*t*) is the normalized fluorescence intensity at time *t*, *I*(*t*) and *I*(0) are the fluorescence intensities of the ROI at times *t* and 0, respectively. *R*(*t*) and *R*(0) are the fluorescence intensities of the reference area at times *t* and 0, respectively. The normalized fluorescence recovery curves were then fitted with a first-order exponential equation (equation (2)) where *A* is the amplitude of the recovery, *τ* is the critical recovery time and *C* is the intercept^55^.

2 $$i\left(t\right)=A\left(1-{e}^{-\frac{t}{\tau }}\right)+C$$

The half-life of the recovery (*t*_1/2_) could then be determined by equation (3) and the apparent diffusion constant (*D*_app_) could be calculated by factoring in the radius of the ROI (*ω*) following equation (4)^55,56^.

3 $${t}_{1/2}=\mathrm{ln}2\times \tau $$

4 $${D}_{{\rm{app}}}=\frac{0.88{\omega }^{2}}{4{t}_{1/2}}$$

### Atomic force microscopy

#### Wet samples for force curve measurements

The freshly cleaved mica was first treated with 20 µM of CaCl_2_ for 20–30 min and washed with water two or three times. The residual solutions were pipetted away from the mica, eventually leaving a thin layer of water on the top. Next, 1 µl of the sample was evenly added onto the mica by immersing the tip of the pipette into the thin water layer. The mica was then incubated at room temperature for at least half an hour. It was either covered or replenished with water to avoid drying during the incubation time. Finally, about 100 µl of the buffer, which contained the same concentration of dextran and PBS as the sample, was mounted onto the mica right before the measurements.

#### Dry samples for tapping mode imaging

Samples were first diluted in water with a final concentration of 10 µM. A total of 5 µl of the diluted sample was immediately spin-coated onto a piece of freshly cleaved mica, of size 1.0 × 1.0–1.5 × 1.5 cm^2^, at 2,000 rpm for 1 min.

#### In situ force curve measurements on tactoids

AFM measurements were conducted on a Cypher Environmental Scanner equipped with a closed gas cell. An active heating and cooling stage was used to actively control the temperature at 20 °C. The measured force curves on the tactoids were recorded in contact mode using a superluminescent diode to reduce the signal-to-noise ratio. Spherical poly methyl methacrylate (PMMA)-based CP-CONT-PM probes (Nanotools, spring constant *k*  =  0.2 N m^−1^; *r* = 750 nm) with a resonance frequency of 30 Hz (approximately 7 Hz in PBS buffer) were used for all measurements. The cantilever was thermally calibrated in solution with blueDrive using the ‘Get Real’ function in the Igor Pro software. Force curves were obtained using a slow scan rate of 0.061 Hz over a force distance of about 4 µm (velocity 414.11 nm s^−1^) to prevent any perturbations in the PBS buffer that could cause the displacing of the tactoids. Furthermore, the trigger point was kept to between 10 and 15 nN. After obtaining the force curves, we applied the Hertz model (for spherical AFM tips) to extract the Young’s modulus of the tactoids (equation (5)).

5 $$F=\frac{4}{3}\times \frac{{E}_{{\rm{s}}}}{1-{V}_{{\rm{s}}}^{2}}\times \sqrt{r}\times {\delta }^{\frac{2}{3}}$$

where *F* is the applied force, *E*_s_ is Young’s modulus, *V*_s_ is the Poisson ratio (*V*_s_ = 0.4), *r* is the radius of the spherical AFM tip and *δ* is the indentation depth. For all samples, several force curves (6–26) were recorded, and mean values and standard deviations were extracted. Two-sample Student’s *t*-tests were performed to verify the difference between two populations.

#### Tapping mode imaging of fibrils

AFM measurements of dry samples in tapping mode (phase less than 90) were similarly recorded on the Cypher Environmental Scanner equipped with a closed cell and a normal laser diode. A heating and cooling stage was used to actively control the temperature at 20 °C. Silicon AC–160TS probes (Oxford instruments, spring constant *k* = 26 N m^−1^; *f* = 300 kHz) with a tip height of 14 µm and a radius of 7 nm were used for all measurements and calibrated using the ‘Get Real’ function in the Igor Pro software. Height images of either 20 × 20 µm or 10 × 10 µm were acquired using a scan rate of 3.0 Hz and 1,024 × 1,024 pixels. Contrast of the images was further enhanced using first-order planefit and flattening using Gwyddion v.2.60. Subsequently, the lengths of the UPy-Gly fibrils in the processed images were measured using the Digimizer software. The histograms of the fibril length were fitted with the Gaussian distributions.

### Cryogenic transmission electron microscopy

For cryoTEM measurements, Quantifoil grids (R 2/2, Quantifoil Micro Tools GmbH) or Lacey grids (LC200-Cu, Electron Microscopy Sciences) were used. Before sample addition, grids were treated with surface plasma at 5 mA for 40 s using a Cressington 208 carbon coater. A total of 3 µl of the sample was applied to the grid held in an automated vitrification robot (FEI Vitrobot Mark IV), operating at 22 °C with a relative humidity of 100%. Excess sample was removed by blotting for 3 s using the filter paper with a blotting force of −2. The thin film formed was vitrified by plunging the grid into liquid ethane just above its freezing point. Vitrified films were transferred into the vacuum of a CryoTITAN (Thermo Fisher) equipped with a field emission gun operated at 300 kV, a postcolumn Gatan energy filter and a 2,048 × 2,048 Gatan CCD camera. Virtrified films were observed in the CryoTITAN microscope at temperatures below −170 °C. Micrographs were taken at low-dose conditions, using defocus values including −10 µm, −5 µm and −2.5 µm at 24,000 magnification.

The fibril diameters were extracted from several cryoTEM images per sample with ImageJ. The histograms of the fibril diameters could be fitted with the Gaussion distribution. Two-sample *t*-tests were carried out to verify the difference of mean values between two populations.

### Polarized fluorescence microscopy

#### Sample preparation

A total of 2 µl of the sample was added to the holding glass and covered with #1.5 cover glass. The sides of the cover glass were sealed with nail polish to avoid evaporation and sample drifting.

#### Optical polarization measurements

The samples were imaged using a commercially available inverted microscope (model: Nikon Eclipse Ti2). After the samples were loaded on the microscope stage, it was illuminated (in epi-illumination mode) using a 637 nm excitation laser (OBIS FP 637LX, Coherent). The excitation parameters (excitation power 0.1 mW) were controlled using the Coherent software. The collimated laser beam was first allowed to pass through a 640 ± 10 nm band-pass filter, followed by a zero-order half-wave plate (Thorlabs) which is mounted on a rotation mount. The excitation beam was reflected by a dichroic mirror (ZT640rdc, Chroma) and was directed to an oil-immersion objective (Apo-TIRF, 60×, 1.49 numerical aperture, Nikon). This set-up results in the sample being illuminated with a collimated light beam with a well-controlled polarization state. To eliminate the concern of the depolarization of the excited light passing through the optics, we mounted a polarizer on top of the objective lens and measured the laser intensity by tuning the angle of the half-wave plate. The minimum laser intensity detected was almost zero. This suggests almost 100% polarization of the excited light.

The fluorescence signal was collected by the same objective lens and passed through a 635 nm notch filter and a 700 ± 75 nm band-pass filter before being collected by the detector (Prime BSI Express sCMOS camera, Teledyne Photometrics). The wide-field fluorescence images from the samples were captured using NIS Elements (Nikon) with an exposure time of 100 ms. For each sample, several 225 s or longer videos were recorded with the half-wave plate manually rotated by 4° every 5 s. This resulted in a polarization rotation of 2*θ* when the half-wave plate was rotated by *θ*.

The fluorescence intensities of the tactoids were extracted using ImageJ and plotted against the polarization angle of the incident light beam (Fig. 3g and Supplementary Fig. 8). To fit the oscillating fluorescence intensity curves, we first studied the bleaching effect and found that the intensities decayed linearly over time and that the bleaching rate was roughly linear to the starting intensity (Supplementary Fig. 6). Subsequently, we modified the squared cosine function and fitted the oscillatory curves with the following equations using Origin.

6 $$I\left(\theta \right)=\left({I}_{0}-{kt}\right){\cos }^{2}\left(\theta +{\theta }_{0}\right)+b\left(1-\frac{{kt}}{{I}_{0}}\right)$$

7 $$t=\frac{5\theta }{8}$$

where *I*_0_ is the amplitude of the oscillation curve and *b* is a constant that is not sensitive to the angle of the linearly polarized light (*θ*). *k* is the decaying rate of the amplitude due to bleaching. *θ*_0_ is related to the orientation of the dyes. The extent of alignment (*α*) of the fibrils within the tactoids can thus be calculated by equation (8).

8 $$\alpha =\frac{{I}_{0}}{{I}_{0}+b}$$

The good correlation between the angles of the dyes (*θ*_0_) and the relative orientations of the tactoids (*θ*_tactoids_) obtained from the images confirmed correlated positioning between the dyes and the fibrils (Supplementary Fig. 7).

### Instant molecular orientation microscopy based on a polarization camera

POLCAM is used to map the molecular orientation distribution in the tactoids^38^. It is a simplified fluorescence orientation microscopy method based on a wide-field fluorescence microscope set-up. In the measurement based on POLCAM, we use the same microscope set-up as in normal linear polarizer-based measurement, as described above, except that instead of an sCMOS camera, we use a polarization camera DZK 33UX250 from The Imaging Source. A quarter-wave plate is installed in the light path to get circularly polarized light for the imaging of the background. The polarization camera is equipped with Sony IMX250MZR Polarsens sensor chip with on-chip polarizers with transmission axis in four directions (0°, 45°, 90° and −45°), thanks to which full Stokes parameters can be calculated on the basis of the fluorescence intensities measured from the four polarization channels. We define the Stokes parameters as follows.

9 $${S}_{0}=\frac{{I}_{0}+{I}_{45}+{I}_{90}+{I}_{135}}{2}$$

10 $${S}_{1}={I}_{0}-{I}_{90}$$

11 $${S}_{2}={I}_{45}-{I}_{135}$$

where *I*_0_, *I*_45_, *I*_90_ and *I*_135_ are measured intensities in the four polarized channels from the polarization camera. Defined as such, the Stokes parameters describe the total intensity of the optical field (*S*_0_), the intensity of linear horizontal or vertical polarization (*S*_1_) and the intensity of linear 45° or −45° polarization (*S*_2_). The pixel-by-pixel Stokes parameters are then used to calculate the in-plane orientation *ϕ* of the fluorescence emission coming from UPy-Cy5 in the tactoids. We define the in-plane orientation of the fluorescence emission by:

12 $$\phi =\frac{1}{2}{\tan }^{-1}\left(\frac{{S}_{2}}{{S}_{1}}\right)={\rm{AoLP}}$$

where AoLP stands for the angle of linear polarization.

To analyse the images from polarization camera measurement, we use the open-source software for the POLCAM method (POLCAM-SR) under the diffraction-limited mode.

### Small-angle X-ray scattering

The SAXS measurements were conducted on a SAXSLAB Ganesha system, equipped with a GeniX-Cu ultra-low divergence source and a photon flux of 1 × 10^8^ photons s^−1^. The wavelength of the X-ray is 1.5 Å. About 150 µl of solutions were loaded into each fixed, 2 mm quartz capillary. Two-dimensional intensity images were collected with a Pilatus 300 K silicon pixel detector with a measurement of 4 h and converted to one-dimensional plots with SAXSGUI. The *q* range covered by a single measurement was 0.12–6.90 nm^−1^ and the absolute *q* value was calibrated with AgBeh. Solvent and capillary contributions to the scattering intensities were subtracted from the blank solution with the PRIMUS program from the ATSAS software package.

### Dynamic and static light scattering

DLS and SLS experiments were performed on an ALV/CGS-3 compact goniometer system (ALV-GmbH), which consists of a single detector, ALV/LSE-5004 light-scattering electronics, several tau digital correlators and a Cobolt Samba 50 laser (laser wavelength 532 nm). DLS/SLS data were collected from 20° to 150° with a 10° step.

## Online content

Any methods, additional references, Nature Portfolio reporting summaries, source data, extended data, supplementary information, acknowledgements, peer review information; details of author contributions and competing interests; and statements of data and code availability are available at 10.1038/s41586-024-07034-7.

### Supplementary information

Supplementary Information Supplementary text and Discussion, Figs. 1–24 and references.

Supplementary Videos Supplementary Videos 1–4 and legends.

## Data Availability

All data needed to evaluate the conclusions in the paper are present in the main text, Supplementary Information and the videos.
