# Supplementary material for: Supramolecular polymers form tactoids through liquid–liquid phase separation
Source: Nature. 2024 Feb 28;626(8001):1011–8. doi: 10.1038/s41586-024-07034-7 (PMC10901743; doi:10.1038/s41586-024-07034-7)
Supplement: Supplementary file 1 — Supplementary text and Discussion, Figs. 1–24 and references. [file 41586_2024_7034_MOESM1_ESM.docx]

Supplementary Information

**Synthesis of UPy-Cy5**


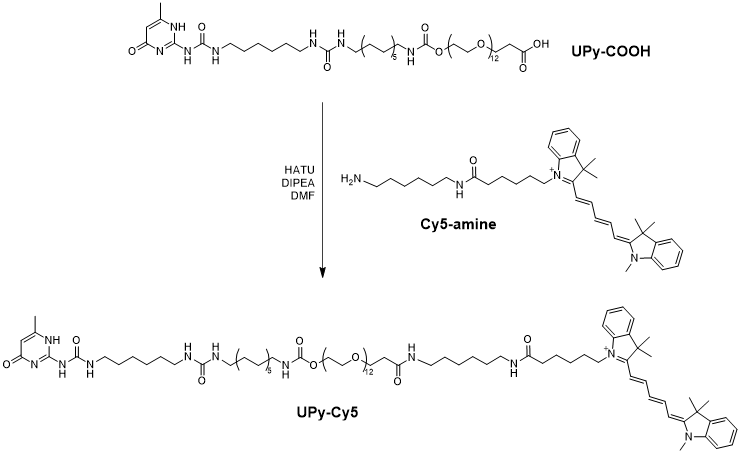


The deprotected UPy-COOH (12 mg; 0.011 mmol) was dissolved in 2 mL of DMF. HATU (5 mg; 0.012 mmol) and DIPEA (9 μL; 0.048 mmol) were then added for pre-activation and the mixture was stirred for five minutes. After that, the Cy5-amine was added (5 mg; 0.0086 mmol) and the reaction was stirred for one hour. The organic phase was then diluted in DCM (10 mL), followed by washing with brine and evaporation of the DCM. The resulting crude was purified by preparative reverse phase LC-MS to yield the desired compound (5.8 mg; 31%). The LC-MS [M] of UPy-Cy5 was calculated 1701.11, found 567.75 [M+4H]^4+^, 851.06 [M+2H]^2+^ and 1701.33 [M+H]^+^.

**Discussions on dynamic and static light scattering spectroscopy (DLS/SLS)**

When the relaxation functions *C(q,t)* in dilute solutions display a single relaxation mode, the full scattered intensity from the solution I(θ) at each observation angle (θ) yields the excess Rayleigh ratio *R(q) = R*_tol_*[(I(θ)- I*_s_*)/I*_tol_*](n*_s_*/n*_tol_*)^2^*, where *I*_s_ and *I*_tol_ are the light scattering intensities from solvent and toluene with refractive indices *n*_s_ and *n*_tol_ and Rayleigh ratio, *R*_tol_ = 2.78 × 10^−5^ cm^−1^. In the Guinier regime (*qR*_g_ < 1), the pattern of *R(q)* is represented by the Zimm equation

*Kc/R(q)=M_w_^-1^(1+2A_2_c)(1+R_g_^2^ q^2^/3)* (S1)

with *R*_g_, *A*_2_ and *c* being the fibril radius of gyration, the second virial coefficient and UPy concentration, respectively, and the optical constant *K= [2πn(dn/dc)]^2^/(N*_A_*λ^4^)*, where *dn/dc* is the refractive index contrast and *N*_A_ is the Avogadro’s number. Eq. S1 applies at low *q*’s yielding *R*_g_ = 72 ± 10 nm for 0 wt% of dextran at the earliest time (0.7 h) to assure dilute conditions, while in the presence of 0.5 wt% of dextran, *R*_g_ is ~10% higher (Extended Data Fig. 3a-c). In this dilute fibril regime, *C(q,t)* displays a single *q^2^* dependent relaxation rate *Γ* yielding the translational diffusion constant of the polymer chain, *D*_f_  *= Γ/q^2^* (in the limit *q* → 0) (Extended Data Fig. 3e) which is related to the fibril hydrodynamic radius *R*_h_ *= k*_B_*T/(6πηD*_f_*)* with *k*_B_, *T* and *η* being the Boltzmann’s constant, temperature and solvent viscosity, respectively. Experimentally, *R*_h_ is found to increase with time and the change is even faster in the presence of 0.5 wt% of dextran, resembling the trend of *R*_g_ (Extended Data Fig. 3e, f).

Assuming similar statistical behavior of the supramolecular fibrils with covalent homopolymers, the values of *R*_h_ and *R*_g_ can be rationalized for rod-like shapes choosing appropriate Kuhn segment length *l*_K_, which is twice the persistence length, *l*_p_^1^. For rigid rods of length *L*, *R*_g_*^2^ = L^2/^12* meaning *L* ~ 250 nm (for *R*_g_ ~ 72 nm), while for semiflexible chains,

$R_{g}^{2}\left( L \right)={Ll}_{K}/3-l_{K}^{2}+\frac{2}{L}l_{K}^{3}-\frac{2}{L^{2}}{\left( 1-e^{-\frac{L}{l_{K}}} \right)l}_{K}^{4}$ (S2)

*l*_K_ ~ 40 nm using *L* = 500 nm. Including polydispersity, with m = (M_w_/M_n_-1)^-1^ = 5 (for M_w_/M_n_ = 1.2) the experimental *R*_g_ is captured with *l*_K_ ~ 60 nm. Complementary information on fibril conformation can be obtained from *R*_h_ (or *D*_f_). In the semi-flexible limit, *L* > 5*l*_K_,

*D_f_ = [kT/(3πηL)][1.843(L/(2l_K_)^0.5^ - log[l/(2l_K_)] - 2.431 - l/d)* (S3)

where *l* (= 0.4 nm) is the space between monomers and *d* (= 4 nm) is the fibril diameter, *l*_K_ ~ 30 nm captures the experimental *D*_f_ . Assuming internal fibril dynamics,

$D_{f}=\frac{k_{B}T}{3\pi\eta L_{w}}*\left( 1+\frac{\sqrt{6}}{\sqrt{\pi}L_{w}}\int_{d}^{l_{p}} \frac{L_{w}-s}{s}Exp\left( \frac{-3d^{2}}{2s^{2}} \right)ds+\int_{l_{p}}^{L_{w}} \frac{L_{w}-s}{\sqrt{2sl_{p}}}Exp\left( \frac{-3d^{2}}{4sl_{p}} \right)ds \right)$ (S4)

where *L*_w_ is the weight-averaged length and *s* is the contour length parameter in the integration. For *L* = 500 nm, *D*_f_ = 5.8$\times$10^-12^ m^2^/s can be captured using *l*_K_ ~ 50 nm.

A model independent estimation of *l*_K_ is obtained from the scattering pattern in a Holtzer representation combining light and SAXS scattering q’s (Extended Data Fig. 3d). *R(q)q* levels-off at *q* ~ 0.03 nm^-1^ identifying the rod-like Kuhn regime (*R(q)~q^-1^* with persistent length ~ 100 nm) whereas at *q* > 0.2 nm^-1^, SAXS probes local packing of fibrils (Fig. 2f). In the regime *qR(q) ~ q^0^*, the limiting *qR/(cK) = πM/L* ~ 8$\times$10^13^ (g/mol)/m), where *M/L* is the mass length density. Assuming 4 pairs of UPy-Gly molecules (1200 g/mol) per rod diameter with inter-disc distance about 0.4 nm, the plateau value *qR/(cK)* amounts to 7.5$\times$10^13^ (g/mol)/m.

In the semi-dilute regime (above the overlap concentration), the translation diffusion constant *D*_f_ of the fibril transforms to the cooperative diffusion of the physically crosslinked network with mesh size *ξ = kT/(6πηD_f_) < R_h_*. With increasing time, *L* increases i.e. *ξ* decreases and *D*_f_ should either increase (good solvent condition) or weakly decrease (bad fibril-solvent interactions). Notably at t > 3 h, *C(q,t)* reveals a second slower diffusion constant, *D*_s_, which could be associated with the self-diffusion of more heterogeneously distributed fibrils^2^. In fact, *D*_s_ compares well with the diffusion of the fibrils in the homogeneous phase (t < 10 h) obtained by FRAP (Fig. 3d) and supports the strong growth of the fibrils at early times.

The LLPS is, rather unexpectedly, not identified by the light scattering probably due to the similar scattering amplitude at *qL* >> 1. Instead, DLS can be sensitive to the crossover to LLPS at t > 10 h (for 0 wt% and 0.5 wt% of dextran) due to the slow tactoid diffusion in the dilute liquid phase as discussed below. FRAP dynamics controlled by fibril diffusion inside tactoids and along the tactoid axis, *D_//_* ≃ 7.5$\times$10^-14^ m^2^/s (0% of dextran and t = 47 h). For rigid rods, *D_//_* ***=*** *[kT/(2πηL)] (δ-γ_1_)* with *δ = ln (2L/d)*, *γ_1_ = 1.27-7.4(1/ δ-0.34)^2^*. Using *η*_water_ = 10^-3^ Pa$\cdot$s, *L* = 2 μm and thickness *d* = 4 nm, *D_//_* ≈ 1.7$\times$10^-12^ m^2^/s. The comparison with the experimental result suggests internal tactoid viscosity ~ 22 times higher than *η*_water_.

Evidence of a LLPS is the presence of an ultra-slow diffusive process in C(q,t), which is resolved for sufficiently long (15, 30 or 60 min) accumulation (Supplementary Fig. 9a,b) for 0 wt% and 0.5 wt% of dextran. The estimated *D*_us_ ≃ 2$\times$10^-14^ m^2^/s **(**0 wt% of dextran at 505 h**)** is associated with tactoid diffusion, *(D^t^_//_ + 2D^t^_┴_)/3* in the dilute liquid phase, where *D^t^_//_ = [kTln (a/b) /(2πηa)]* with *a*, *b* being the length of the long and short axis of tactoids, and *D^t^_┴_ = D^t^****_//_*** */2*. Assuming a water like viscosity and prolate ellipsoid shape (Fig. 3f), *D*_us_ corresponds to tactoid dimensions *a* ≃ 30 μm and *b* ≃ 4 μm, which is consistent with the aspect ratio of tactoids in the presence of 0.5 wt% of dextran (~ 6.5).

**Flory-Huggins Solution Theory**

$f_{Mix}=\frac{\varphi}{N}ln\varphi+\left( 1-\varphi\right)\ln\left( 1-\varphi\right)+\chi\varphi(1-\varphi)$ (S5)

The Flory-Huggins mixing free energy per unit volume, for a polymer with a degree of polymerization N, can be written in the dimensionless form above (Eq. S5), where φ is the volume fraction of the polymer (0 < φ <1) and χ is the polymer-solvent interaction parameter. The first two terms represent the entropy and the last term stands for the enthalpy. To have phase separation,$f_{Mix}$is supposed to be positive. Since the entropic terms are negative, χ must be a positive value, suggesting that UPy-Gly supramolecular polymers and water have repulsive interactions. For reference, χ between PEG and water is ~0.3^3^. For a small χ, the sign of $f_{Mix}$could be switched from negative to positive with the increase of N or the elongation of supramolecular polymers, which means a transition from a stable solution to a metastable or unstable solution. In the metastable state (binodal region), the phase separation proceeds through the nucleation and growth pathway. In the unstable state (spinodal region), the solution would undergo spinodal decomposition. The slow growth of the tactoids especially at relatively low dextran concentrations (Extended Data Fig. 4) suggests a nucleation and growth pathway or a metastable state of the solution.

**Macromolecular crowder effect on the phase separation**

$f_{Mix}=\frac{\varphi_{1}}{N_{1}}ln\varphi_{1}+\frac{\varphi_{2}}{N_{2}}ln\varphi_{2}+\varphi_{s}ln\varphi_{s}+\chi_{12}\varphi_{1}\varphi_{2}+\chi_{1s}\varphi_{1}\varphi_{s}+\chi_{2s}\varphi_{2}\varphi_{s}$ (S6)

Based on the Flory-Huggins solution theory, the mixing free energy per unit volume, for two polymers with degrees of polymerization as N_1_ and N_2_, can be written in the dimensionless form above (Eq. S6), where $\varphi_{1}, \varphi_{2}, \varphi_{s}$ are the volume fractions of polymer 1, polymer 2 and the solvent ($\varphi_{1}+ \varphi_{2}+ \varphi_{s}=1$). $\chi_{12}, \chi_{1s}, \chi_{2s}$ are the interaction parameters of polymer 1/polymer 2, polymer 1/solvent and polymer 2/solvent, respectively^4^. For dextran, its interaction parameter with water is ~0.5 ($\chi_{2s})$at 25 ℃^5^. Since UPy-Gly supramolecular polymers and dextran could phase separate from each other, $\chi_{12}$ is also supposed to be positive. When the dextran concentration ($\varphi_{2}\ll1$) is increased, $f_{Mix}$ would also increase. This means that a smaller N_1_ could also make $f_{Mix}$ positive or that the phase separation of supramolecular polymers could occur with shorter fibril lengths in the presence of higher concentrations of dextran ($\varphi_{1}\ll1, \varphi_{2}\ll1)$. Based on the tactoids of amyloid fibrils, the longer the fibril length, the higher the axial ratio. The decrease of axial ratios above 0.9 wt% of dextran could be related to the enhanced phase separation tendency at shorter fibril lengths. At 3 wt% of dextran and above, the solution turned turbid immediately after mixing, similar to spinodal decomposition.

**LLPS of UPy-COOH and BTA-EG_4_**

Similar to UPy-Gly, UPy-COOH supramolecular polymers can also undergo LLPS and form into tactoids, whose shapes and sizes can be controlled by the dextran concentration (Extended Data Fig. 9b). BTA-EG_4_, however, needs to be coupled with a charged surfactant to induce the formation of tactoids (Extended Data Fig. 9a). The surfactant can be inserted into the supramolecular polymer chains by hydrophobic interactions as reported by our previous work^6^. The charges introduced by the surfactants could reduce fibril entanglement and increase the fibril mobility for easier LLPS. But too many surfactants would make the fibrils too short and/or too repulsive to each other to phase separate.

**Continuous and discrete networks of UPy-Gly and UPy-COOH supramolecular polymers**

Both UPy-Gly and UPy-COOH are charged, and their zeta potentials are dependent on the pH and salt concentrations (Supplementary Fig. 4). When 5 wt% of PEG or dextran was introduced into the solution of UPy-COOH supramolecular polymers (50 µM), clear difference could be seen using CLSM at different PBS concentrations and pH (Supplementary Fig. 21-22). The aggregation tendency increased at higher PBS concentrations and lower pH due to reduced repulsive interactions. Similarly, in the phase separated solution of PEG and dextran, the supramolecular polymers stayed liquid like at relatively high pH and/or low salt concentrations, which would first undergo LLPS at the liquid-liquid interface and eventually form into a continuous network. At low pH and/or high salt concentrations, the supramolecular polymers turned into rigid rods almost immediately in the PEG/dextran solution and formed into discrete networks that supported or deformed the droplets (Supplementary Fig. 24).

**Supplementary Figures**


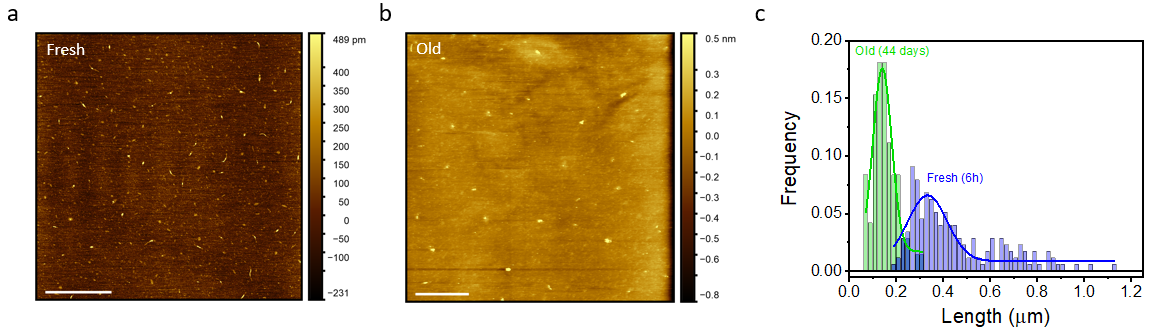


**Supplementary Fig. 1 | Comparison of the fresh and old UPy-Gly fibrils at 6 hours after being broken.** a, Representative AFM image for fresh fibrils (6 hours old). The scale bar is 4 µm. b, Representative AFM image for old fibrils (44 days old). The scale bar is 2 µm. c, Histograms of the fresh and old fibril lengths 6 hours after being broken. n = 177 (fresh) and 72 (old). The average lengths are 0.45 and 0.15 um for fresh and old fibrils, respectively. This suggests that fresh fibrils grew faster than old ones if we assume similar length distributions for fresh and old samples right after the shearing. UPy-Gly 1 wt% (8.4 mM), pH = 7.6, PBS $\times$ 0.25.

**
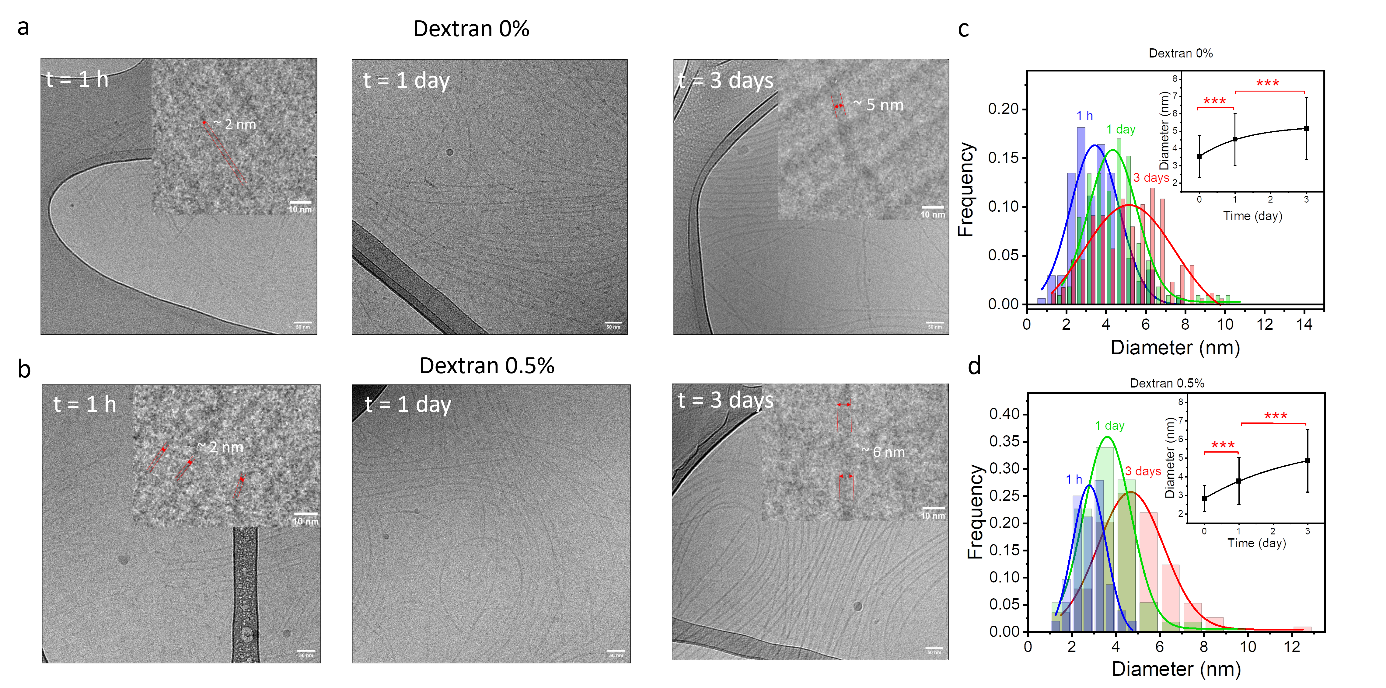
**

**Supplementary Fig. 2 | Tracking the change of fibril diameters with Cryo TEM.** a-b, Representative cryo TEM images for the solution with 0 wt% (a) and 0.5 wt% (b) of dextran. The scale bars for the insets and the large pictures are 10 nm and 50 nm, respectively. c-d, Histograms of the fibril diameter distributions at t = 1 h, 1 day and 3 days for the samples with 0 wt% of dextran (c), n = 112 to 176 and 0.5 wt% of dextran (d), n = 104 to 168. Other solution conditions: UPy-Gly 1 wt%, pH = 7.6, PBS $\times$ 0.25. ***: p < 0.01.


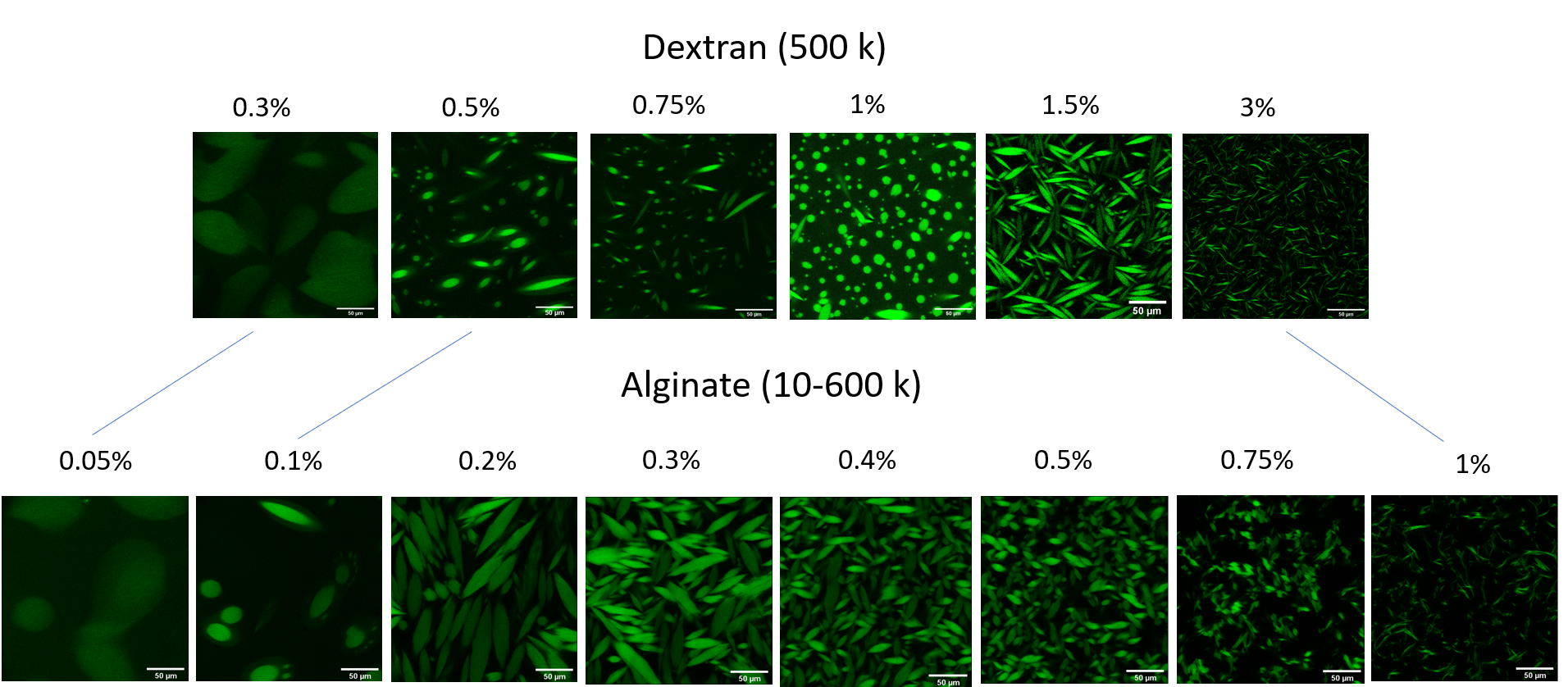


**Supplementary Fig. 3 | Comparison of the exclusion effect of dextran and alginate in the LLPS of UPy-Gly supramolecular polymers.** Top panel: UPy-Gly solutions with different concentrations of dextran. Bottom panel: UPy-Gly solutions with different concentrations of alginate. All the percentages listed in the image referred to weight percentages. Solution condition: UPy-Gly 1 wt%, pH = 7.6 or 7.7, PBS $\times$ 0.25.


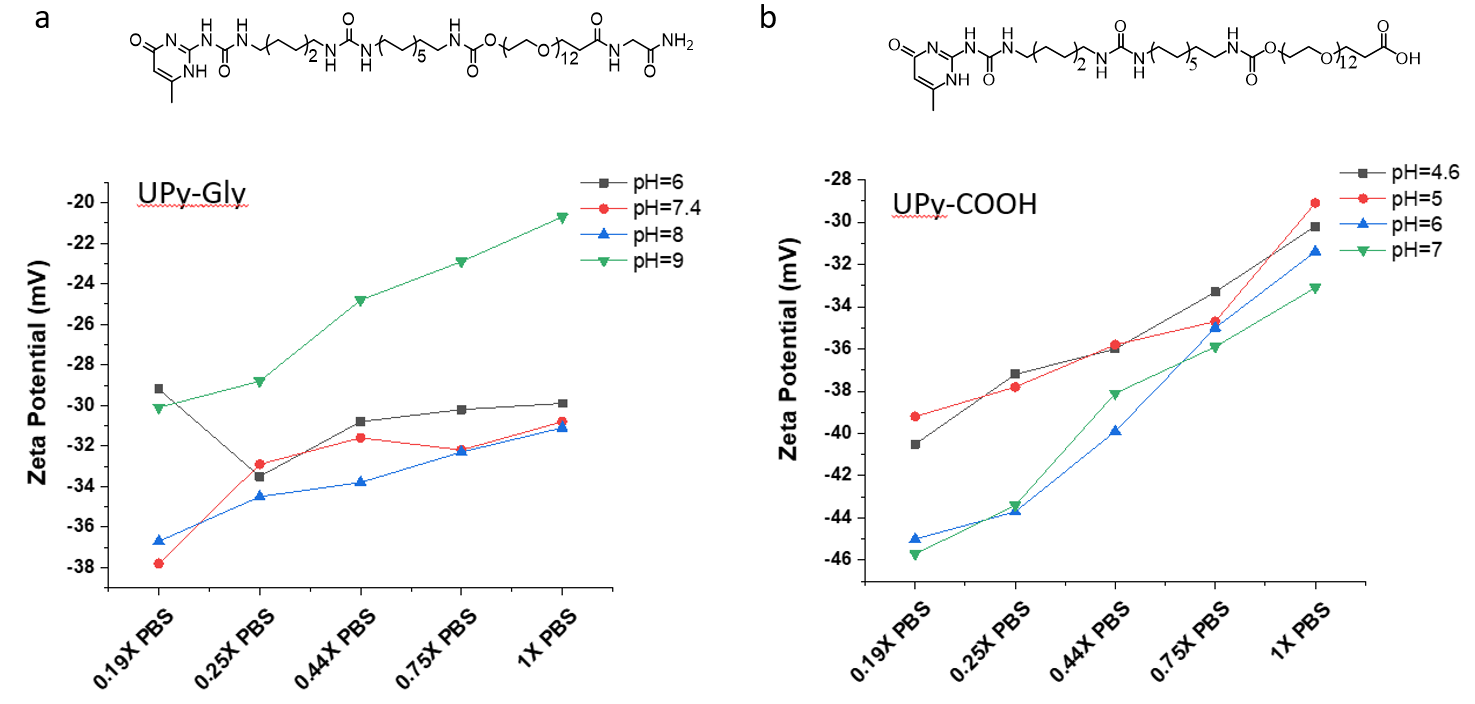


**Supplementary Fig. 4 |** Zeta potentials of UPy-Gly (a) and UPy-COOH (b) as a function of pH and PBS concentrations.

| 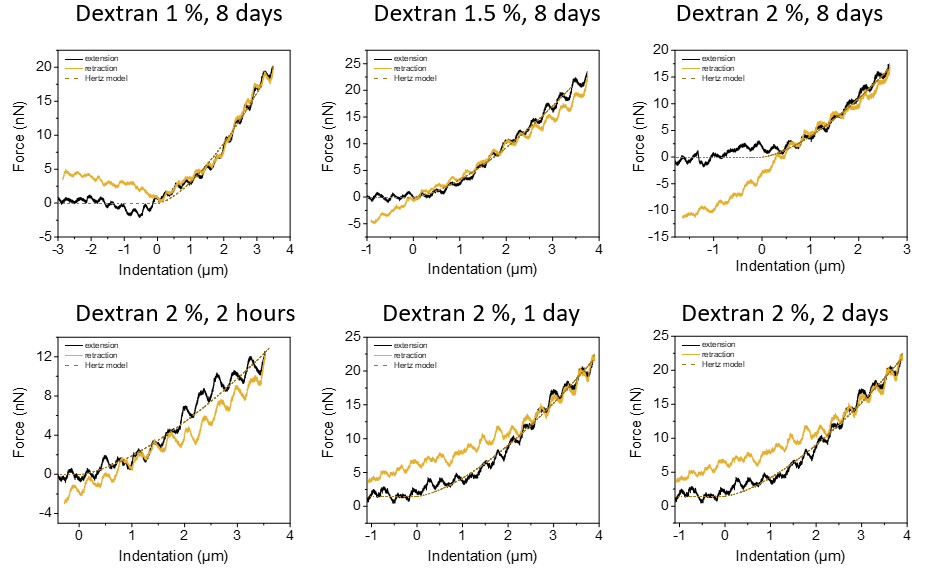 |
| --- |

**Supplementary Fig. 5 | Representative AFM force curves for tactoids with different ages and different dextran concentrations.** Black line: extension curve, yellow line: retraction curve, dashed line: fitted lines of the extension curves with Hertz model. Solution condition: UPy-Gly 1 wt%, pH = 7.6, PBS $\times$ 0.25, Dextran 1 wt%, 1.5 wt% and 2 wt%.


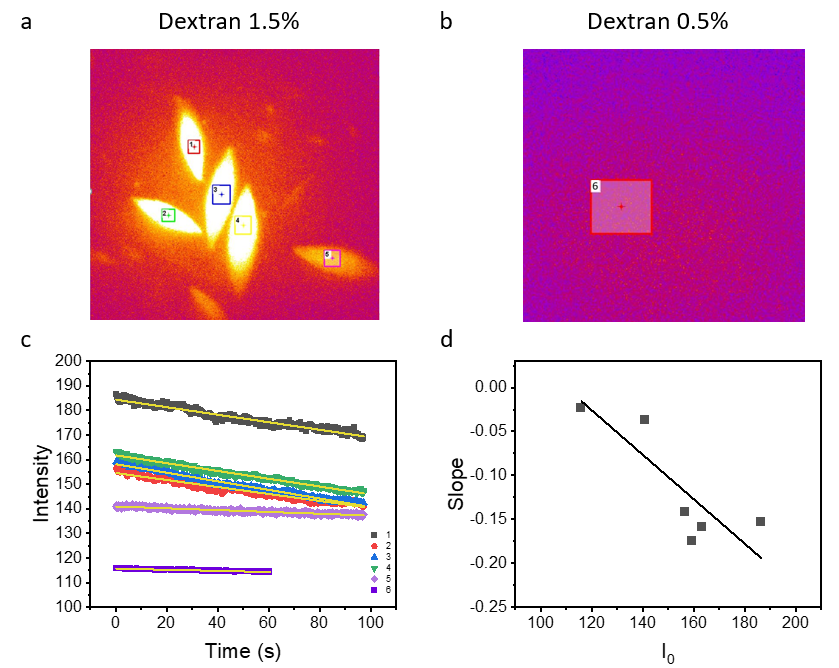


**Supplementary Fig. 6 | Linearly decaying fluorescence intensities due to bleaching.** a-b, Representative images from polarized fluorescence microscope for solutions with 1.5 wt% of dextran after phase separation (a) and 0.5 wt% of dextran before phase separation (b). Solution condition: UPy-Gly 1 wt%, pH = 7.6, PBS $\times$ 0.25. c, Plot of fluorescence intensities over time for the selected areas in a and b. d, Plot of the fluorescence intensity decaying slopes against the starting intensity at the selected areas in a and b.


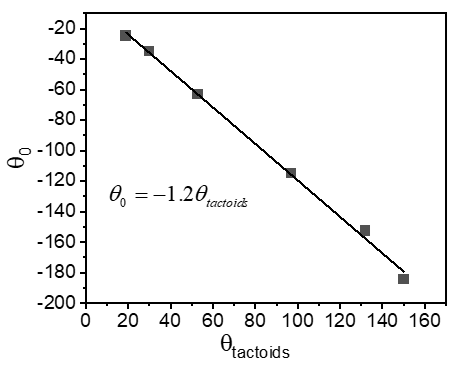


**Supplementary Fig. 7 | Plot of the relative angle of the dyes (θ_0_) against the angle of the tactoids (θ_tactoids_).** Solution condition: UPy-Gly 1 wt%, Dextran 1 wt%, pH = 7.5, PBS $\times$ 0.25, 1 day old.


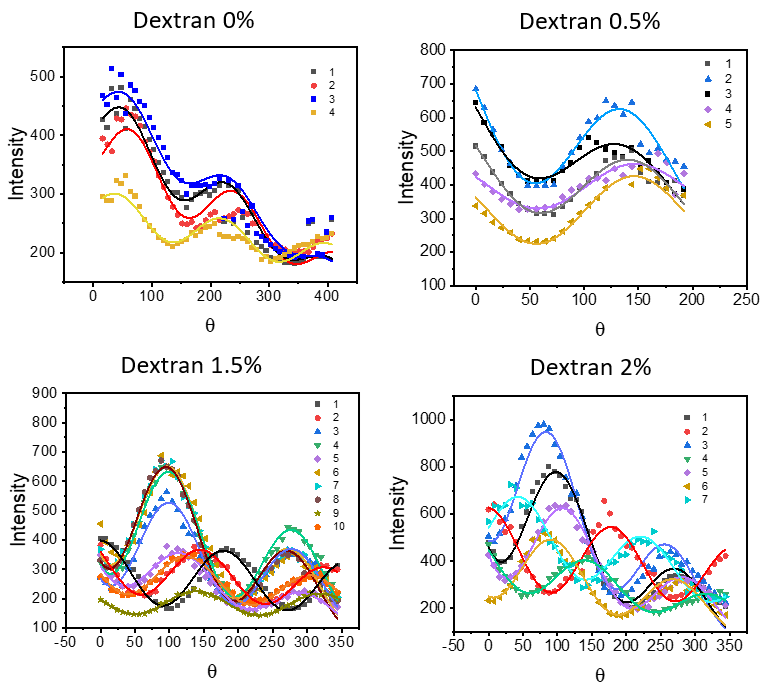


**Supplementary Fig. 8 | Polarised fluorescence microscope image analysis for tactoids in the presence of different concentrations of dextran.** Example fluorescence intensity oscillation curves and fitted lines. The numbers in the legends refer to different positions on the tactoids. Solution condition: UPy-Gly 1 wt%, Dextran: 0 wt%, 0.5 wt%, 1.5 wt% and 2 wt%, pH = 7.5, PBS $\times$ 0.25, 2 days old.

**
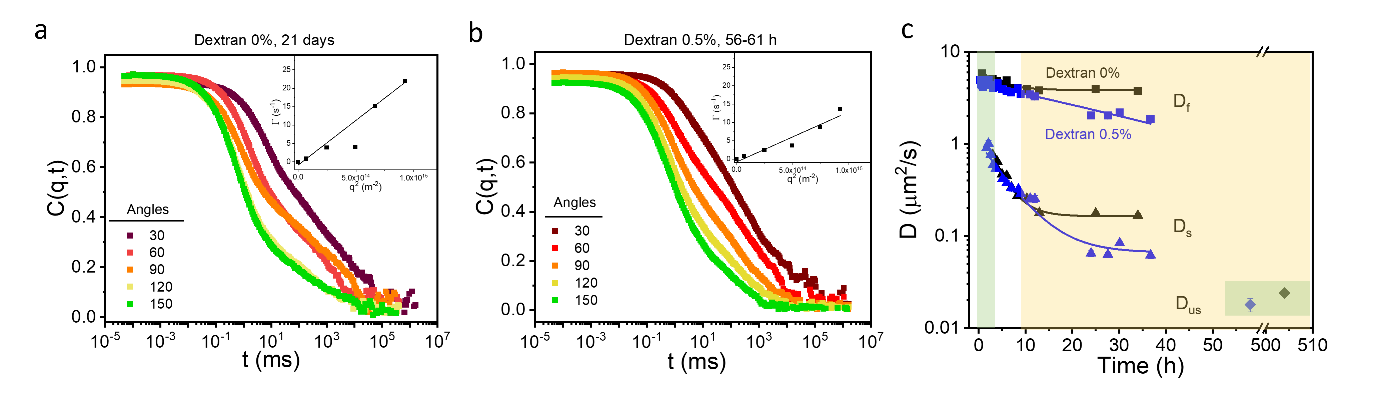
**

**Supplementary Fig. 9** | Relaxation function *C(q,t)* of the concentration fluctuation in LLPS regime (late times) with 1 wt% of UPy-Gly and 0 wt% (a) and 0.5 wt% (b) of dextran at different scattering angles i.e., *q*’s. The slowest diffusion coefficient, *D*_us_, is indicated by the *q^2^* dependent relaxation rate, *Γ* (insets) obtained from the inverse-Laplace-transformation of the experimental *C(q,t)*. c, The different diffusion coefficients obtained from the inverse-Laplace-transformation of the experimental *C(q,t)* in the fibril solutions with 0% (black) and 0.5% (blue) of dextran. In the dilute regime (green shaded area), the fast *D*_f_ ~ 4 to 6 μm^2^ /s represents the translation diffusion of single fibrils, yielding *R*_h_. Crossover to semidilute regime (overlap of fibrils) (white and yellow area), *D*_f_ becomes cooperative diffusion controlled by the mesh size of the fibril network, and a new slower, *D*_s_ represents the self-diffusion of the fibrils^2^. *D*_s_ drops with time due to increasing length and solution viscosity in particular at early time (*L* increases from 0.51 $\pm$ 0.21 μm at t = 15 min to 1.70 $\pm$ 1.12 μm at t = 8 h for the solution with 0% of dextran according to AFM) where the increase rate is significant, whereas it levels-off above ~ 10 h (yellow shaded area) in agreement with Fig. 2c. The scattering from the tactoids due to their tens of μm size is documented by the presence of an ultra-slow diffusion constant, *D*_us_, describing the tactoid diffusion in the dilute liquid phase. This ultra slow process should be present at t > 10 h but it was verified at much longer annealing time to enable sufficiently long accumulation (15 min, 30 min or 1 h) of *C(q,t)*.


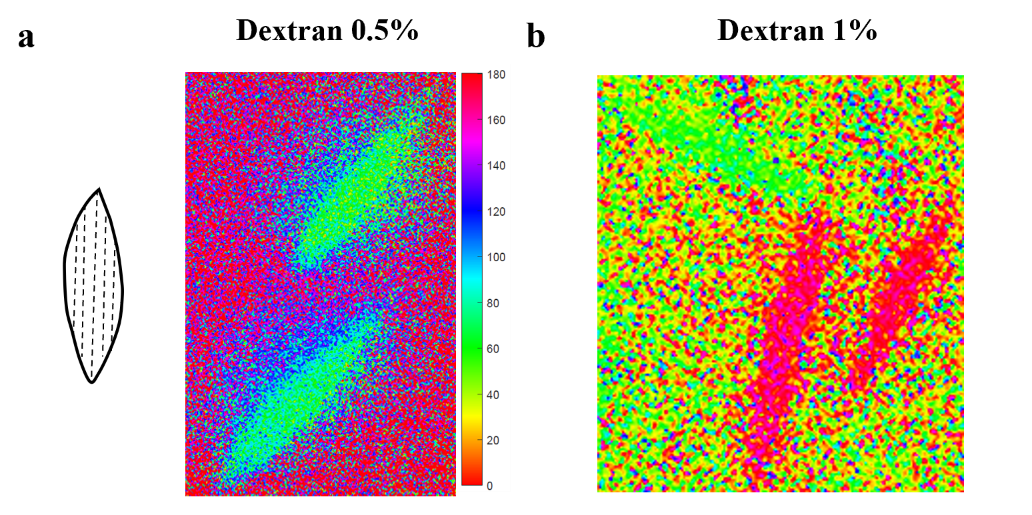


**Supplementary Fig. 10 | POLCAM images for tactoids with 0.5 wt% (a) and 1 wt% (b) of dextran.** Solution condition: UPy-Gly 1 wt%, pH = 7.6, PBS $\times$ 0.25, 1 day old.


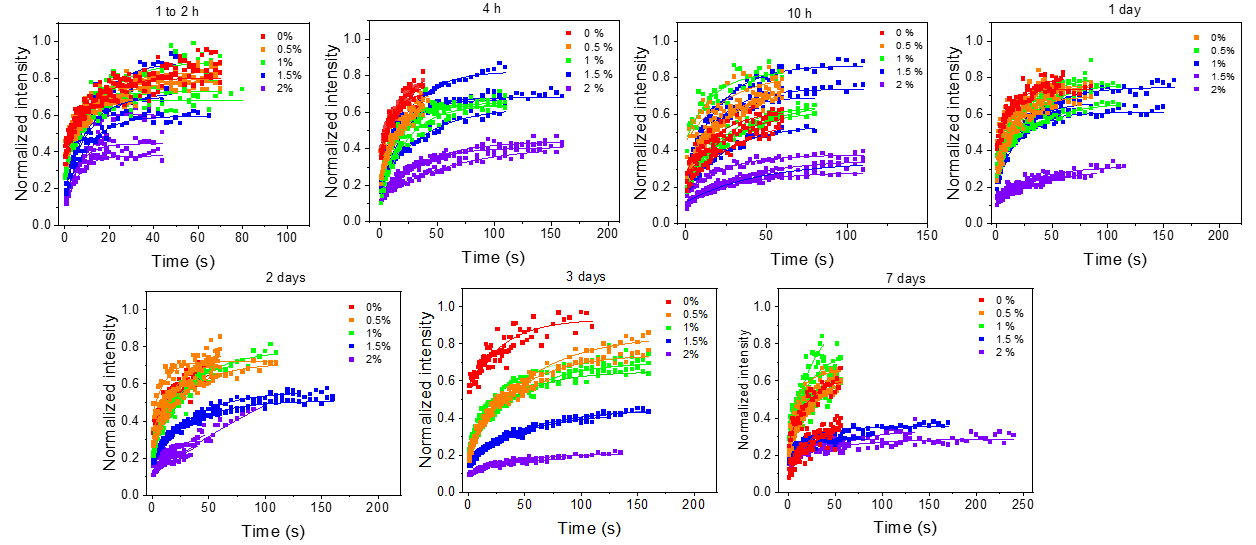


**Supplementary Fig. 11 | FRAP curves and fitted lines for different solutions at different time.** Solution condition: UPy-Gly 1 wt%, Dextran: 0 wt%, 0.5 wt%, 1 wt%, 1.5 wt%, 2 wt%, pH = 7.6, PBS $\times$ 0.25. Time: 1 to 2 hours, 4 hours, 10 hours, 1 day, 2 days, 3 days, and 7 days.


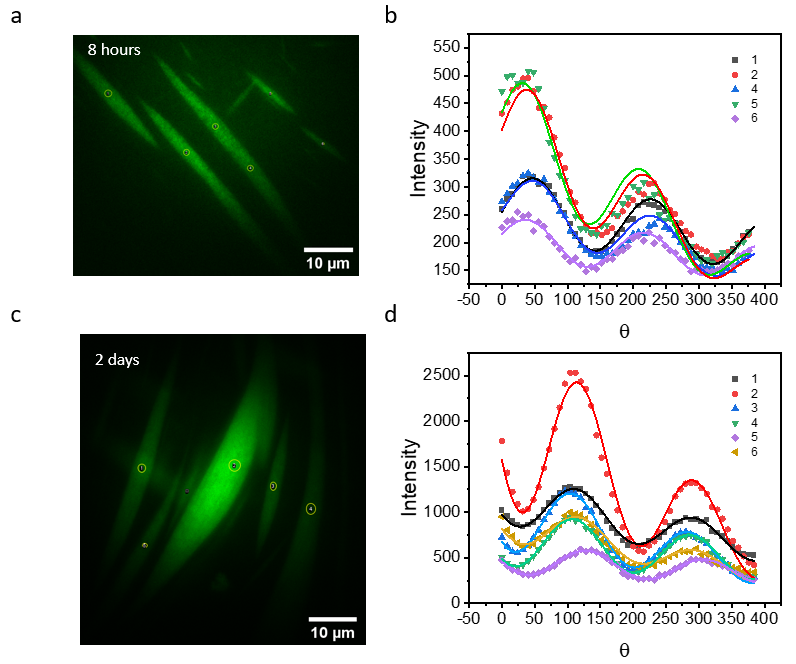


**Supplementary Fig. 12 | Polarised fluorescence microscope image analysis for tactoids at different time.** a, Representative image for 8 hours old of tactoids. b, Fitting of the fluorescence intensity oscillation curves extracted from a. c, Representative image for the 2 days old tactoids. d, Fitting of the fluorescence intensity oscillation curves extracted from c. Solution condition: UPy-Gly 1 wt%, Dextran 1 wt%, pH = 7.5, PBS $\times$ 0.25.


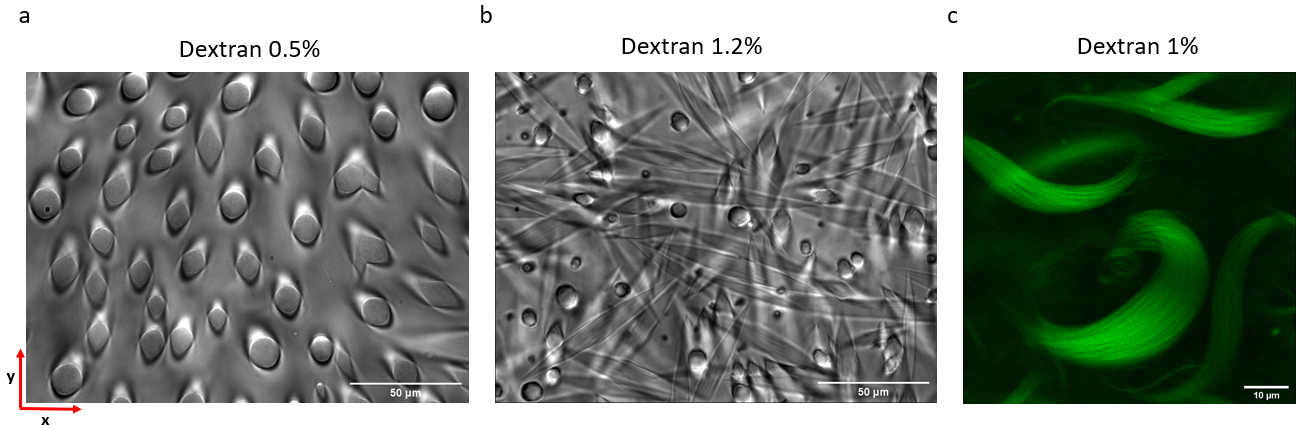


**Supplementary Fig. 13 | Bundling of fibrils within the tactoids.** a-b, Tactoids visualized by bright field imaging at the x-y plane with 0.5 wt% (a) and 1.2 wt% (b) of dextran. c, Confocal image of tactoids with 1 wt% of dextran (1 month old). The tactoids were treated with acetone through slow solvent exchange. Solution condition: UPy-Gly 1 wt%, pH = 7.6, PBS $\times$ 0.25.


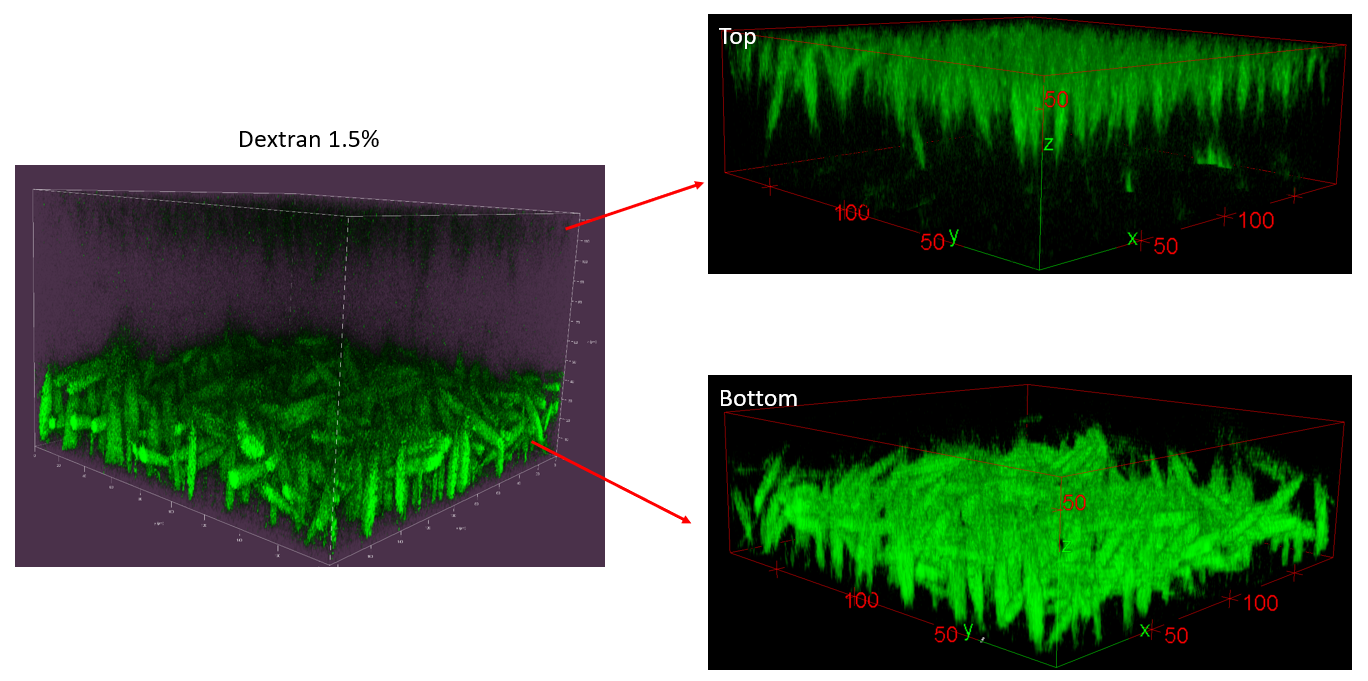


**Supplementary Fig. 14 | 3D images of tactoids with 1.5 wt% of dextran.** Solution condition: UPy-Gly 1 wt%, Dextran 1.5 wt%, pH = 7.6, PBS $\times$ 0.25, 14 days. The sample was incubated in a 120 μm thick chamber with one piece of glass on the top and at the bottom each. The unit of the xyz axis is μm.


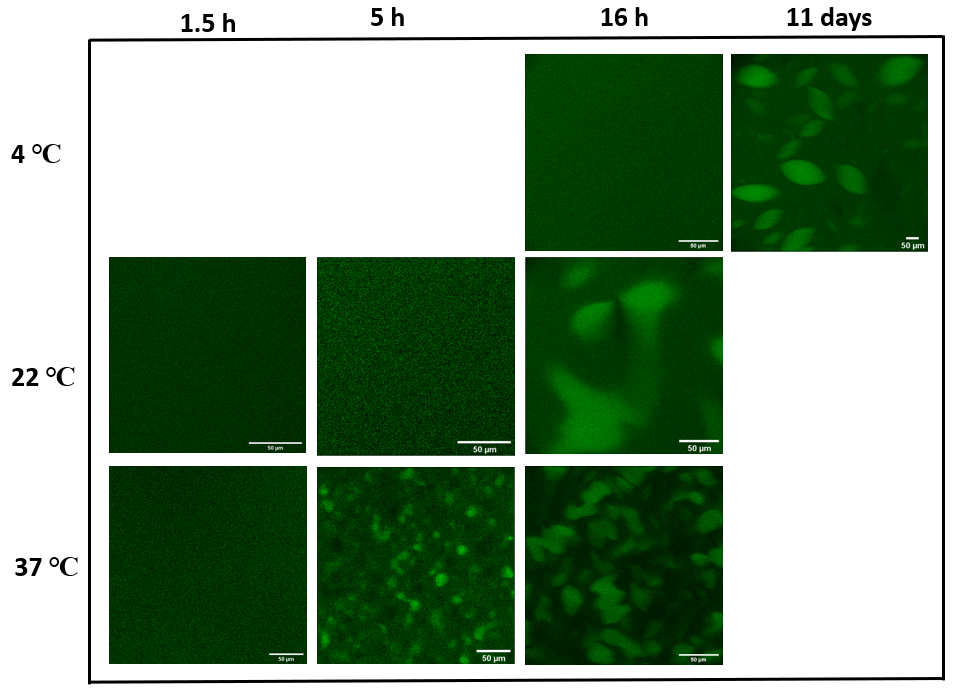


**Supplementary Fig. 15 | Temperature effect on the kinetics of LLPS of UPy-Gly supramolecular polymers.** The LLPS became faster in the order of 4 ℃ (top row), 22 ℃ (middle row) and 37 ℃ (bottom row). The solutions were imaged at 1.5 h, 5 h, 16 h and 11 days, respectively. Solution condition: UPy-Gly 1 wt%, Dextran 0 wt%, pH = 9, PBS $\times$ 0.25. Scale bar is 50 μm.

**
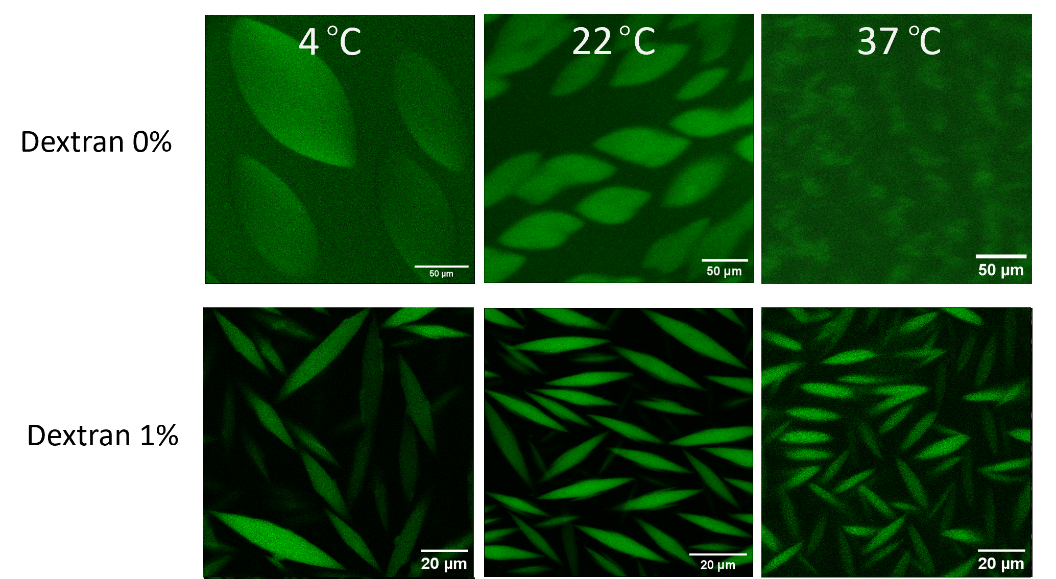
**

**Supplementary Fig. 16 | Temperature effect on the morphology of tactoids formed by the LLPS of UPy-Gly supramolecular polymers.** The tactoids become smaller and less ordered at higher temperature. Solution condition: UPy-Gly 1 wt%, pH = 7.7, PBS $\times$ 0.25, Dextran 0 wt% (top row) and 1 wt% (bottom row), temperature 4 ℃ (left), 22 ℃ (middle) and 37 ℃ (right). The scale bar is 50 μm in the top row and 20 μm in the bottom row.


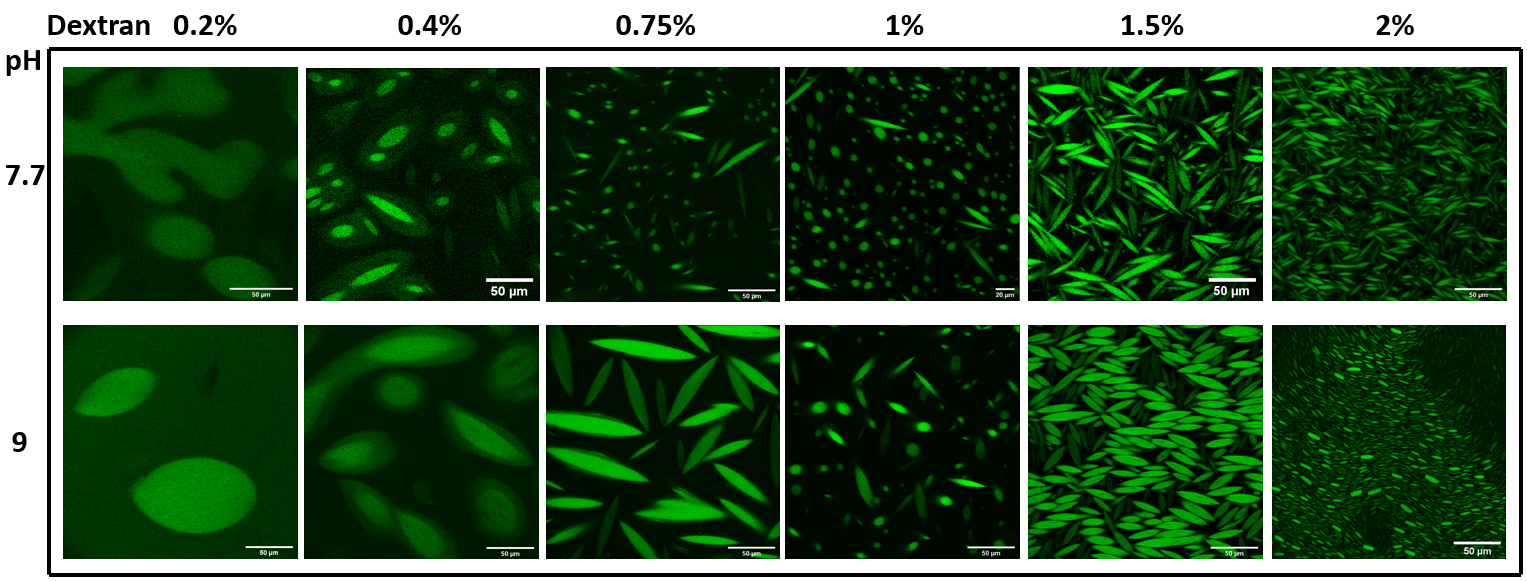


**Supplementary Fig. 17 | The influence of pH and dextran concentration on the morphology of tactoids.** Solution condition: UPy-Gly 1 wt%, PBS $\times$ 0.25, pH = 7.7 (top row, 2 days old), 9 (bottom row, 14-22 hours), Dextran 0.2 wt%, 0.5 wt%, 0.75 wt%, 1 wt%, 1.5 wt%, 2 wt%. The scale bar is 20 μm for the solution with 1 wt% of dextran at pH = 7.7. The scale bar is 50 μm for the rest.


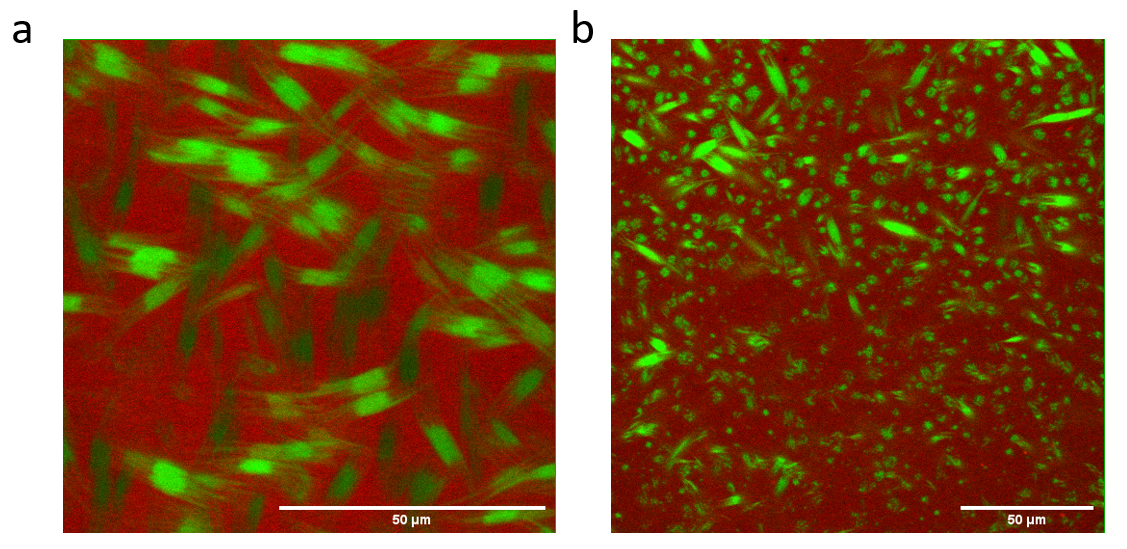


**Supplementary Fig. 18 | Salt effect on the morphology of tactoids.** a pH = 9, PBS $\times$ 0.25, b pH = 8.8, PBS $\times$ 0.44. Solution condition: UPy-Gly 1 wt%, Dextran 2 wt%, 8 days old. Green : UPy-Cy5, red: Dextran-FITC. Scale bar: 50 μm. The circular structures in b are cross sections of the vertically standing tactoids.


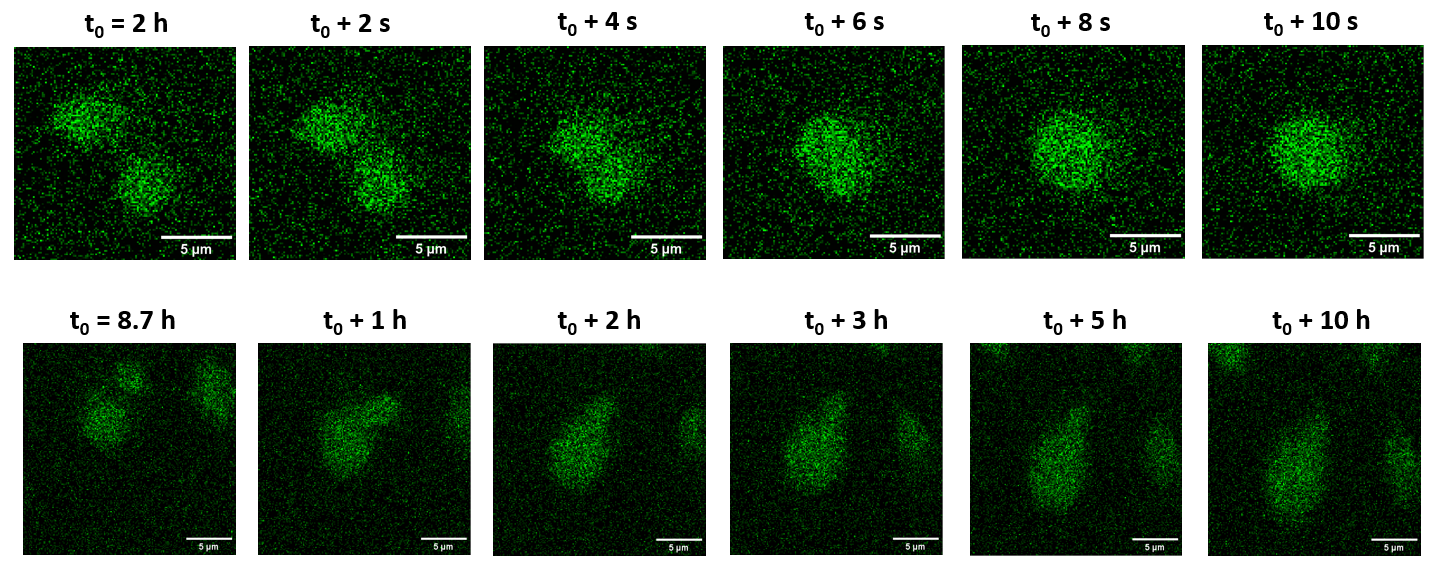


**Supplementary Fig. 19 | Fusion of vertically standing tactoids at different aging time.** At t_0_ = 2 h, the vertically standing tactoids could be merged in a few seconds, leaving a round cross section. At t_0_ = 8.7 h, the fusion of the tactoids slows down dramatically, leaving an irregularly shaped cross section. Solution condition: UPy-Gly 1 wt%, Dextran 1.5 wt%, pH = 7.6, PBS $\times$ 0.25.


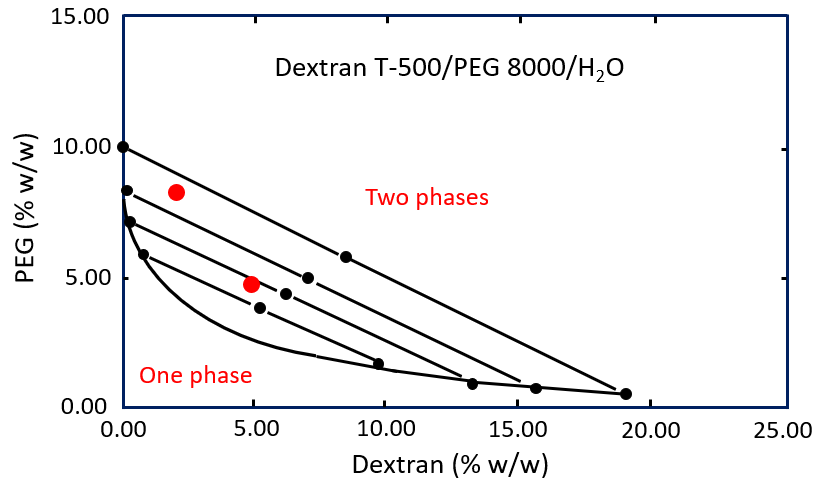


**Supplementary Fig. 20 | Phase diagram of PEG (8 kDa) and dextran (T-500) in aqueous solution**^7^**.** The red dots represent the compositions used in this paper, which are 5 wt% PEG/ 5 wt% dextran and 8 wt% PEG /2 wt% dextran.

**
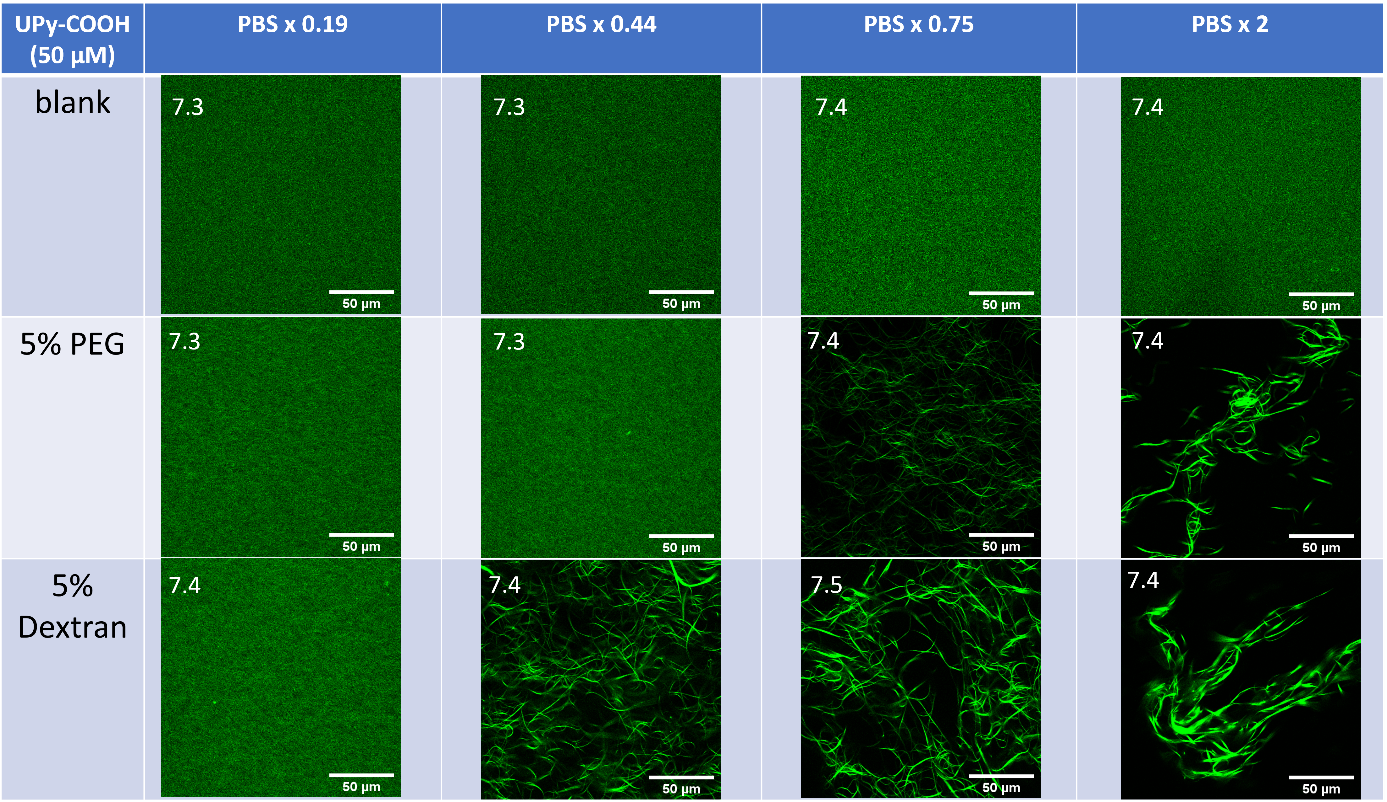
**

|  |
| --- |
| **Supplementary Fig. 21 \| Salt effect on the aggregation of UPy-COOH supramolecular polymers.** Solution conditions: UPy-COOH 50 µM, pH = 7.3 to 7.5, PBS $\times$ 0.19, 0.44, 0.75, 2. Top row: blank, middle row: + 5 wt% of PEG, bottom row: + 5 wt% of dextran. t < 1 day. |

**
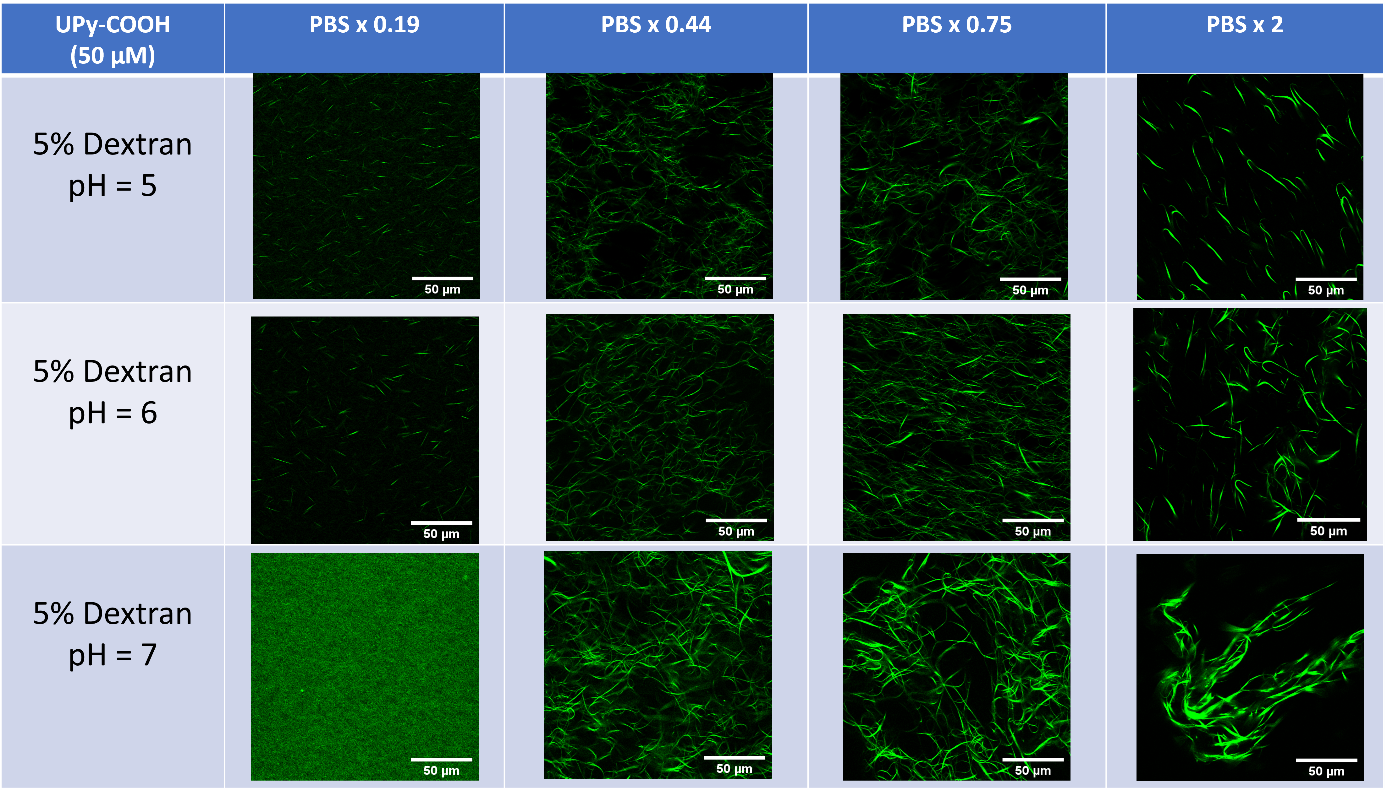
**

**Supplementary Fig. 22 | pH effect on the aggregation of UPy-COOH supramolecular polymers.** Solution conditions: UPy-COOH 50 µM, 5 wt% of dextran, pH =5, 6, 7, PBS $\times$ 0.19, 0.44, 0.75, 2. t < 1 day.

**
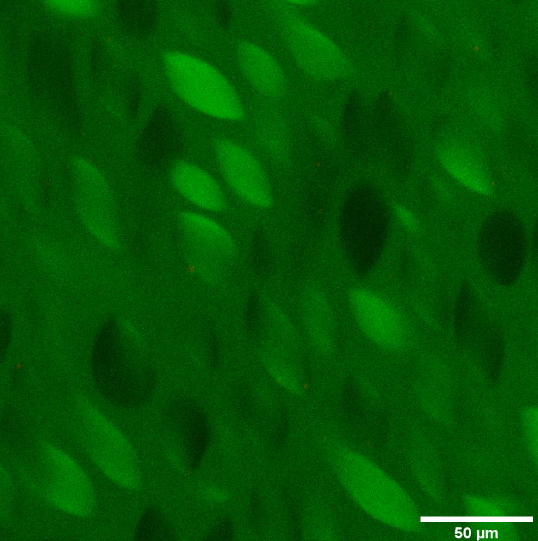
**

**Supplementary Fig. 23 | Tactoids formed in the PEG phase after removal of dextran droplets through centrifugation.** Solution condition before centrifugation: 1 wt% of UPy-Gly, 5 wt% of PEG, 5 wt% of dextran, pH = 9, PBS $\times$ 0.25, 0.5 μM Nile Red, t = 9 h. The picture was taken for the upper layer of the solution after centrifugation.

| a | 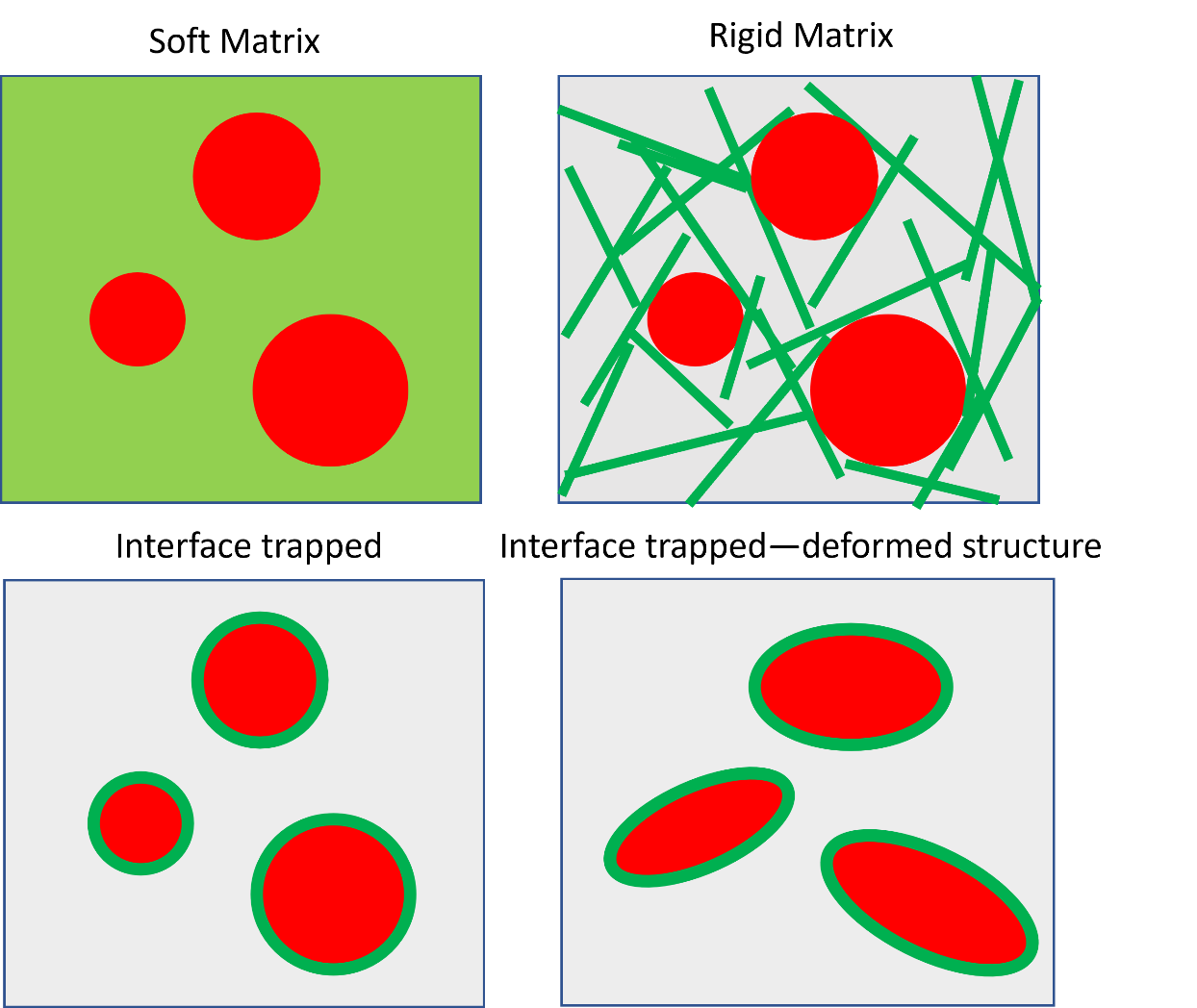 |
| --- | --- |
|  |  |
| b | 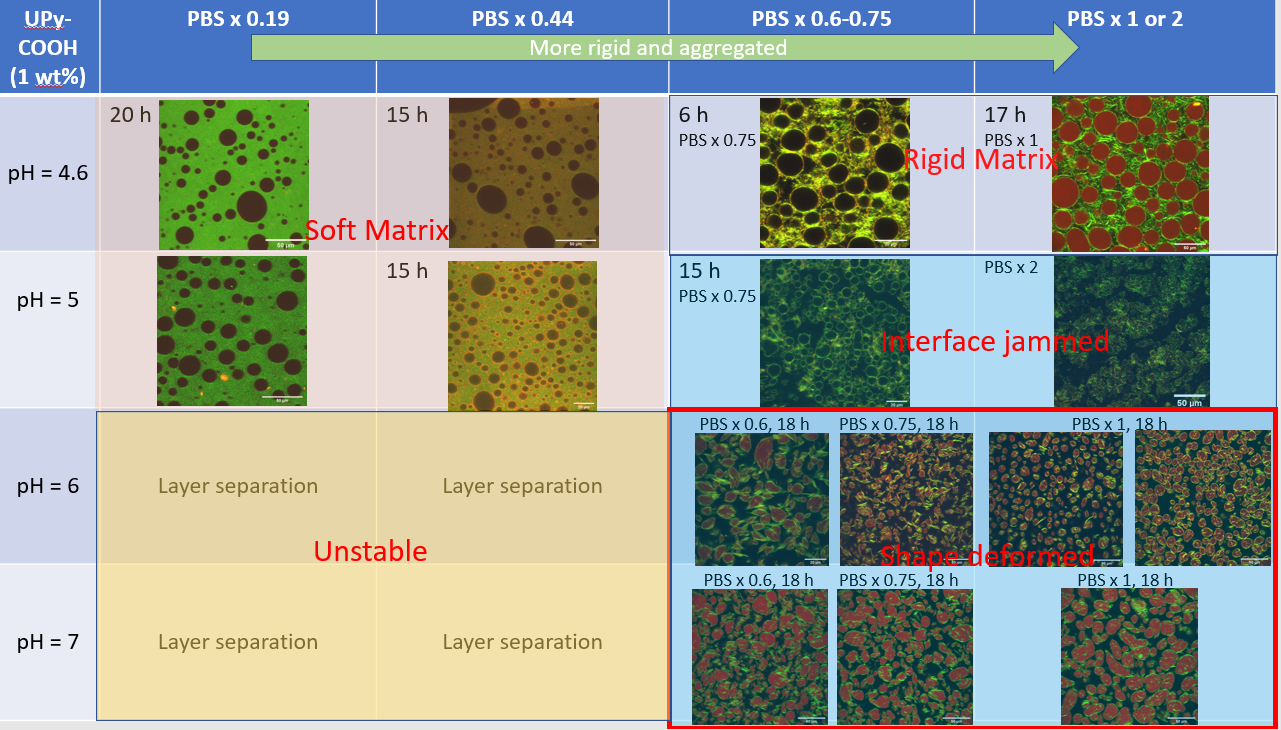 |

**Supplementary Fig. 24 | pH and salt effect on the supramolecular network in the PEG/dextran solution.** a. Schemes of droplets in the supramolecular networks. b. CLSM images for the dextran droplets supported by the supramolecular networks. Solution condition: 1 wt% UPy-COOH, 5 wt% PEG and 5 wt% dextran, pH = 4.6, 5, 6 and 7, PBS $\times$ 0.19, 0.44, 0.6, 0.75, 1 and 2, aging time 15 to 20 h. UPy-COOH is labelled by UPy-Cy5 or nile red (green). Dextran is labelled by dextran-FITC (red). The fluorescence intensity of FITC is rather low at pH = 4.6 and 5, leading to dark droplets. The scale bar is 50 μm.

**References**

1. Dutertre, F. *et al.* Structure and dynamics of dendronized polymer solutions: Gaussian coil or macromolecular rod? *Macromolecules* **49**, 2731-3740 (2016).

2. Jian, T. *et al.* Dynamics of composition fluctuations in diblock copolymer solutions far from and near to the ordering transition. *Macromolecules* **27**, 4762-4773 (1994).

3. Bae, Y. C., Shim, J. J., Soane, D. S. & Prausnitz, J. M. Representation of vapor–liquid and liquid–liquid equilibria for binary systems containing polymers: applicability of an extended Flory–Huggins equation. *J. Appl. Polym. Sci.* **47**, 1193-1206 (1993).

4. Shrinivas, K. & Brenner, M. P. Phase separation in fluids with many interacting components. *Proc. Natl. Acad. Sci. USA* **118**, e2108551118 (2021).

5. Bercea, M., Nichifor, M., Eckelt, J. & Wolf, B. A. Dextran-Based polycations: Thermodynamic interaction with water as compared with unsubstituted dextran, 2-flory/huggins interaction parameter. *Macromol. Chem. Phys.* **212**, 1932-1940 (2011).

6. Su, L. *et al.* Dilution-induced gel-sol-gel-sol transitions by competitive supramolecular pathways in water. *Science* **377**, 213–218 (2022).

7. Diamond, A. D. & Hsu, J. T. Phase diagrams for dextran-PEG aqueous two-phase systems at 22°C. *Biotechnology Techniques* **3**, 119-124 (1989).
